# Switching to the cyclic pentose phosphate pathway powers the oxidative burst in activated neutrophils

**DOI:** 10.1038/s42255-022-00550-8

**Published:** 2022-03-28

**Authors:** Emily C. Britt, Jorgo Lika, Morgan A. Giese, Taylor J. Schoen, Gretchen L. Seim, Zhengping Huang, Pui Y. Lee, Anna Huttenlocher, Jing Fan

**Affiliations:** 1 Morgridge Institute for Research, Madison, WI USA; 2 Department of Nutritional Sciences, University of Wisconsin-Madison, Madison, WI USA; 3 Cell and Molecular Biology Graduate Program, University of Wisconsin-Madison, Madison, WI USA; 4 Department of Medical Microbiology and Immunology, University of Wisconsin-Madison, Madison, WI USA; 5 Comparative Biomedical Sciences Graduate Program, University of Wisconsin-Madison, Madison, WI USA; 6 Division of Immunology, Boston Children’s Hospital, Harvard Medical School, Boston, MA USA; 7 Department of Pediatrics, University of Wisconsin-Madison, Madison, WI USA

**Keywords:** Metabolomics, Innate immune cells, Metabolism

## Abstract

Neutrophils are cells at the frontline of innate immunity that can quickly activate effector functions to eliminate pathogens upon stimulation. However, little is known about the metabolic adaptations that power these functions. Here we show rapid metabolic alterations in neutrophils upon activation, particularly drastic reconfiguration around the pentose phosphate pathway, which is specifically and quantitatively coupled to an oxidative burst. During this oxidative burst, neutrophils switch from glycolysis-dominant metabolism to a unique metabolic mode termed ‘pentose cycle’, where all glucose-6-phosphate is diverted into oxidative pentose phosphate pathway and net flux through upper glycolysis is reversed to allow substantial recycling of pentose phosphates. This reconfiguration maximizes NADPH yield to fuel superoxide production via NADPH oxidase. Disruptions of pentose cycle greatly suppress oxidative burst, the release of neutrophil extracellular traps and pathogen killing by neutrophils. Together, these results demonstrate the remarkable metabolic flexibility of neutrophils, which is essential for their functions as the first responders in innate immunity.

## Abstract (web summary)

Upon activation, neutrophils undergo rapid metabolic reconfigurations towards pentose cycling, which powers their effector functions.

## Main

Neutrophils are the most abundant leukocytes in the circulation, with a crucial role in innate immunity. Upon sensing signals associated with infection, neutrophils can rapidly turn on a series of effector functions, including the production of reactive oxygen species (ROS), the release of antibacterial peptides and proteases and the release of neutrophil extracellular traps (NET), to eliminate pathogens^1–3^. ROS have a central role in neutrophil functions, not only by acting as key pathogen-killing agents but also through activation of granular proteases and induction of NET release^4–6^. The rapid production of ROS upon activation is termed oxidative burst, which is catalysed by a multicomponent enzyme, NADPH oxidase (NOX)^7^. Impaired oxidative burst due to NOX mutations causes chronic granulomatous diseases, which leave patients highly susceptible to infections^8,9^, highlighting the importance of oxidative burst in innate immunity.

Emerging studies have demonstrated that specific metabolic rewiring is often coupled to immune response to both support and orchestrate immune functions^10–14^ in T cells, macrophages, natural killer cells and so on^15–17^. Compared to that of other immune cells, understanding of neutrophil metabolism is only just beginning^18–20^. As short-lived cells at the first line of immune defence, neutrophils have the unique ability to turn on antipathogen responses very quickly. Because such rapid activation of effector functions is associated with substantial metabolic demands, a critical aspect of neutrophil metabolism is to understand how they utilize limited metabolic resources to support the considerable increase in metabolic demand within a short time. For instance, within 10–30 min after stimulation, neutrophils can engage in oxidative burst where oxygen consumption rate rises >30-fold. This requires a large amount of NADPH to drive the reduction of oxygen. Many previous studies have investigated this key question: what metabolic strategies do different cells use to fulfil the NADPH demand associated with different biological processes? Various pathways, including pentose phosphate pathway (PPP)^21–24^, malic enzyme^25,26^, isocitrate dehydrogenase^27,28^ and folate pathways^29,30^, have been reported to contribute significantly under different situations (for example, biosynthesis during proliferation, lipogenesis, redox defence and so on). Among these, upregulation of oxidative PPP (oxPPP) has been found particularly important in supplying NADPH during acute oxidative stress^21,31–33^. Like the oxidative burst (where NADPH is required to drive the production of ROS from oxygen), defence against acute oxidative stress (where NADPH is required to drive the reduction in ROS to less reactive products like H_2_O) is also associated with a sudden, profound increase in NADPH demand. Studies in acute oxidative stress suggest that PPP has high potential capacity to supply NADPH on demand^22,34^. Recent studies in various immune cells have additionally indicated the importance of PPP in immune functions^35–37^, including some in neutrophils suggesting the dependence of oxidative burst on glucose metabolism or oxPPP^38–40^. However, the specific metabolic reprogram that rapidly powers up neutrophil effector functions upon activation has not been quantitatively revealed.

Here we characterized the rapid metabolic changes in neutrophils activated by a series of stimuli, and identified that a common feature specifically coupled to oxidative burst is the substantial rewiring of PPP. During oxidative burst, oxPPP is greatly upregulated and becomes the dominant pathway for glucose metabolism. At the same time, net flux through upper glycolytic enzyme glucose-6-phosphate isomerase (GPI) is reversed. This forms the unique metabolic mode of pentose cycle, where overflowing pentose produced by oxPPP is converted by non-oxidative PPP (non-OxPPP) and reversed GPI back to glucose-6-phosphate (G6P), which then re-enters oxPPP. Pentose cycle enables ultra-high NADPH yield (up to six NADPH per glucose) that greatly exceeds what is known in other mammalian cells. Notably, the switch to pentose cycle upon activation is rapid, on demand, and quantitatively coupled to the strength of oxidative burst across various types and doses of stimulation. Specific inhibition of oxidative burst in stimulated neutrophils resets cells to using glycolysis-dominant glucose metabolism. Disruption of pentose cycle by inhibition of oxPPP or genetic knockout (KO) of key non-OxPPP enzymes strongly inhibits oxidative burst, NET release and pathogen killing. This discovery reveals the impressive and unique metabolic flexibility in neutrophils that is essential for their function in innate immunity.

## Results

### Neutrophils rapidly reprogram metabolism upon stimulation

**Fig. 1:**
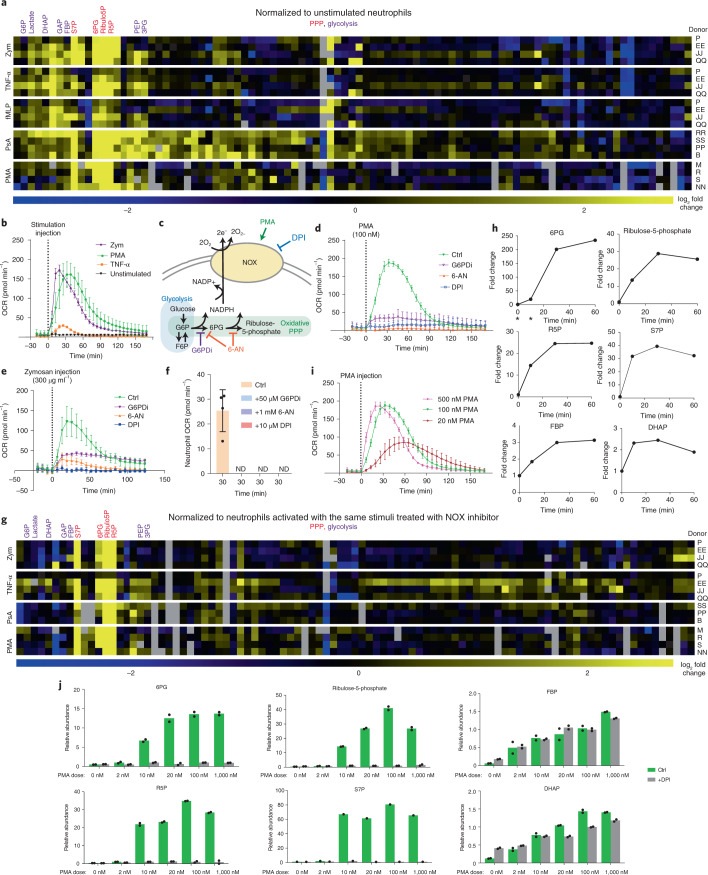
Rapid changes in PPP coupled to oxidative burst. **a**, Heatmap showing metabolomic changes in human neutrophils activated ex vivo with zymosan (zym; 300 µg ml^–1^, 30 min), TNF-α (100 ng ml^–1^, 30 min), fMLP (100 nM, 10 min), PsA (10:1 PsA/neutrophils, 30 min) or PMA (100 nM, 30 min). Colour represents fold change compared to unstimulated neutrophils from the same donor. **b**, Oxidative burst induced by zymosan (500 µg ml^–1^), TNF-α (100 ng ml^–1^) or PMA (100 nM) (mean ± s.d., *n* = 6 technical replicates). **c**, NOX, stimulated by PMA and inhibited by DPI, produces superoxide using NADPH as the electron donor. OxPPP produces NADPH and can be inhibited by G6PDi or 6-AN. **d**,**e**, Oxidative burst induced by PMA (**d**) or zymosan (**e**) is inhibited by G6PDi, 6-AN or DPI (mean ± s.d., *n* = 6 (PMA), *n* = 8 (zymosan) technical replicates). **f**, Oxygen consumption by neutrophils incubated with PsA for 30 min with or without treatment by 6-AN, G6PDi or DPI (mean ± s.d., *n* = 4 technical replicates, indicated by dots). ND, not detectable (OCR for neutrophils not significantly different from zero). **g**, Heatmap showing metabolomic differences in human neutrophils stimulated ex vivo with zymosan (300 μg ml^–1^), PMA (100 nM), TNF-α (100 ng ml^–1^) or PsA for 30 min with or without DPI (10 µM) treatment. Colours indicate relative metabolite levels in conditions without DPI treatment compared to corresponding conditions (same donor, same stimulant) with DPI treatment. **h**, Changes in PPP and glycolytic intermediates over time upon stimulation with 100 nM PMA. Relative level is normalized to unstimulated neutrophils. *, Below reliable quantitation. Centre of the plot shows mean value from technical duplicates. **i**, Oxidative burst induced by varying PMA doses (mean ± s.d., *n* = 6 technical replicates). **j**, Relative abundances of PPP and glycolytic metabolites at 30 min after stimulation with varying doses of PMA. Bar graphs represent mean values of technical replicates (indicated by individual dots). **b**,**d**–**f**,**h**–**j**, Results confirmed in at least two independent experiments using neutrophils from different donors. **a**,**g**, Results from different donors presented in different rows. Ctrl, control. Source data

To systematically characterize metabolic alterations associated with neutrophil activation, we examined the response in human peripheral blood neutrophils stimulated ex vivo with five different stimuli: (1) zymosan A, a fungal wall component, (2) tumour necrosis factor (TNF)-α, an inflammatory cytokine, (3) N-formylmethionyl-leucyl-phenylalanine (fMLP), a bacterial peptide analogue, (4) *Pseudomonas aeruginosa* (PsA), a bacterial pathogen and (5) phorbol myristate acetate (PMA), a protein kinase C activator. Metabolomic analysis revealed substantial and rapid (within 10 or 30 min) alterations that were largely consistent across donors (Fig. 1a). Interestingly, we observed that these five stimuli, which activate different receptors and targets, induced many similar metabolic changes. Most notably, all detected intermediates in the PPP accumulated profoundly and many intermediates in glycolysis increased to a lesser extent, pointing to a substantial rewiring of these pathways that is commonly induced upon activation.

### PPP rewiring is specifically coupled to oxidative burst

**Extended Data Fig. 1:**
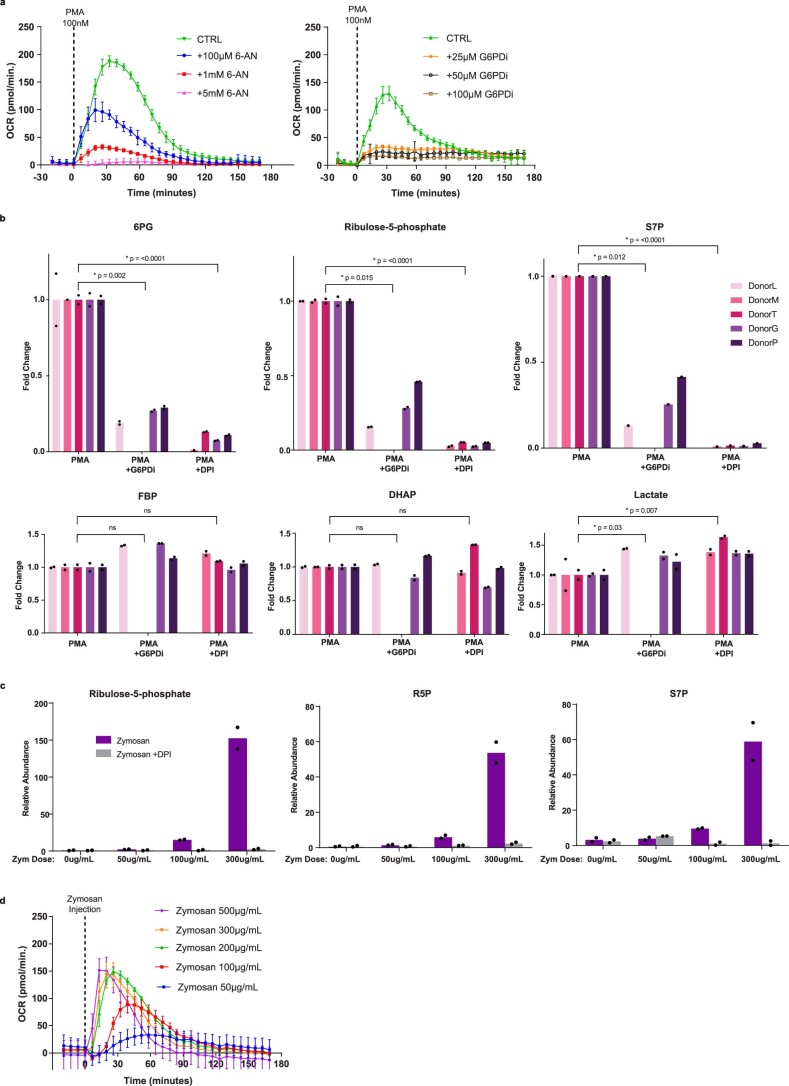
Oxidative burst relies on oxidative PPP, and the rewiring of PPP is coupled to the oxidative burst (Related to Fig. 1). a. The oxidative burst induced by PMA (100 nM) is inhibited by varying concentrations of 6-AN or G6PDi. (Mean ± SD, N = 6 technical replicates). b Relative levels of PPP intermediates 6PG, ribulose-5-phosphate, S7P, and glycolytic intermediates: FBP, DHAP, and lactate 30 minutes after 100 nM PMA stimulation, with or without the treatment of G6PDi (50 µM) or DPI (10 µM). Measurements were made in neutrophils isolated from 5 donors, as indicated by color codes. (Neutrophil yield from every donor was not always high enough to cover all three conditions, so N = 5 PMA, N = 4 PMA + DPI, N = 3 PMA + G6PDi). Relative level is normalized to neutrophils stimulated with 100 nM PMA without inhibitor from the same donor. Bar graph indicates mean value of two technical replicates, as indicated by dots. Statistical significance was determined by paired, two-tailed t-test across biological replicates (mean values of technical replicates per donor), with p-values indicated in the figure c. Dose-dependent changes in the levels of PPP intermediates in human peripheral blood neutrophils stimulated with various doses of zymosan with or without DPI (10 µM). Bar graphs show mean value of technical duplicates indicated by dots. d. Oxidative burst induced by various doses of zymosan A, Mean ± SD, N = 6 technical replicates. Source data

The five stimuli examined can activate a diversity of effector functions; one common function rapidly induced by all is oxidative burst^41,42^. Oxidative burst is quantitively indicated by increase in NOX-dependent oxygen consumption rate, which peaks around 30 min after stimulation by zymosan, PMA or TNF-α (Fig. 1b). Oxidative burst requires a large amount of NADPH as the reducing power (Fig. 1c). OxPPP is known as a major pathway supplying cellular NADPH in many biological contexts^23^. Inhibition of oxPPP using the inhibitors glucose-6-phosphate dehydrogenase (G6PDi) or 6-aminonicotinamide (6-AN) can suppress oxidative burst induced by various stimuli nearly as complete as direct inhibition of the NOX complex with diphenyleneiodonium chloride (DPI) (Fig. 1d–f), and the inhibition of oxidative burst by oxPPP inhibitor is dose-dependent (Extended Data Fig. 1a). These results, consistent with previous reports^38,39^, suggest that the activation of oxidative burst is dependent on oxPPP.

Interestingly, we found that substantial rewiring of PPP is also dependent on the activation of oxidative burst. To isolate the metabolic changes directly associated with oxidative burst from those generally induced by stimulation, we compared the metabolomic profiles of neutrophils stimulated with zymosan, TNF-α, PsA or PMA for 30 min, with or without treatment with the NOX inhibitor DPI (Fig. 1g). While many stimulation-induced changes across the metabolic network persisted regardless of DPI treatment, this analysis highlighted that the upregulation of PPP intermediates is specifically dependent on NOX activity. Furthermore, we found accumulation of PPP intermediates closely correlated with the strength of oxidative burst over time and across varying stimulation dosage. Upon stimulation with 100 nM PMA, intermediates in PPP and glycolysis accumulate very rapidly and reached a plateau when oxidative burst reached its peak (by ~30 min; Fig. 1h). With increasing dosage of PMA, oxidative burst became faster and stronger, reaching a maximum at ~100 nM (Fig. 1i). PPP intermediates also increased in a dose-dependent manner and reached a maximum (>10-fold) at ~100 nM. Such PPP accumulation can be completely prevented by NOX inhibition at any PMA dose (Fig. 1j). In contrast, glycolytic intermediates such as fructose-1,6-bisphosphate (FBP) and dihydroxyacetone phosphate (DHAP) also increased in a PMA dose-dependent manner, but such increase was largely not prevented by NOX inhibition (Fig. 1j and Extended Data Fig. 1b). These results demonstrate that PMA-induced rewiring of PPP, but not glycolysis, is fully dependent on oxidative burst.

Similarly, we observed dose-dependent accumulation of PPP intermediates that correlates with dose-dependent increase in oxidative burst upon zymosan stimulation (Extended Data Fig. 1c,d). Together, these data illustrate interdependence between the activation of oxidative burst and substantial rewiring of PPP. Such interdependence is specific among the broader range of metabolic alterations and functional changes associated with neutrophil activation induced by various stimuli.

### PPP becomes the major metabolic route during oxidative burst

**Fig. 2:**
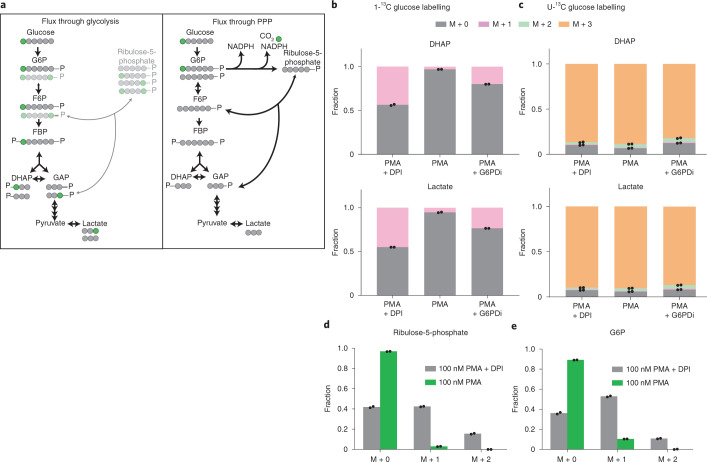
Neutrophils switch to use of oxidative PPP as the dominant route for glucose metabolism during PMA-induced oxidative burst. **a**, Schematic illustrating major expected isotopomers when 1-^13^C-glucose is fully metabolized via glycolysis (left) or oxidative PPP (right). Some isotopomers generated from reversible activity are shown in grey shading. **b**,**c**, Labelling pattern of DHAP and lactate from 1-^13^C-glucose (**b**) and U-^13^C-glucose (**c**) in PMA-stimulated neutrophils. **d**,**e**, Labelling pattern of ribulose-5-phosphate (**d**) and glucose-6-phosphate (**e**) from 1-^13^C-glucose in stimulated neutrophils with or without oxidative burst. M+0 indicates fraction of the compound with unlabeled molecular mass, M+1 indicates fraction of the compound with 1 heavy labeling (one ^13^C-labelled carbon), and so on. Bar graph represents mean of *n* = 2 technical replicates, indicated by dots. Trends were confirmed in two independent experiments using cells from different donors. Source data

Interdependence between the activation of oxidative burst and rewiring of PPP suggests that neutrophils probably redirect glucose into oxPPP to increase NADPH supply. To quantitively measure the redistribution of glucose metabolism flux coupled to oxidative burst, we performed a tracing study with 1-^13^C-glucose in neutrophils stimulated with 100 nM PMA for 30 min, with or without DPI treatment. OxPPP specifically releases the C1 carbon of G6P as CO_2_, while glycolysis retains this carbon. Therefore, when 1-^13^C-glucose is metabolized via glycolysis it yields 50% unlabelled and 50% 1-labelled three-carbon lower glycolytic metabolites (such as DHAP and lactate); alternatively, when 1-^13^C- glucose is metabolized via oxPPP, labelling would be lost in downstream metabolites (Fig. 2a)^43^. We found that, without oxidative burst, DHAP and lactate were close to 50% labelled, indicating that glycolysis is the major route for glucose metabolism. However, during PMA-induced oxidative burst, this labelled fraction dropped sharply to <5%. Treatment of G6PDi, which partially inhibits oxPPP, partially prevented the loss of labelling (Fig. 2b). To verify whether loss of labelling is specific to the C1 position rather than a result of preferential usage of non-glucose substrates, we performed parallel U-^13^C-glucose labelling. Unlike 1-^13^C-glucose tracing, U-^13^C-glucose resulted in near-complete labelling (~90%) of lower glycolytic intermediates under all three conditions (Fig. 2c), suggesting that PMA-stimulated neutrophils rely mainly on extracellular glucose. Together, these data show that oxidative burst drives a switch from glycolysis to oxPPP as the dominant route for glucose metabolism in PMA-stimulated neutrophils.

### Neutrophils switch to cyclic PPP to maximize NADPH yield

We next quantitatively examined the mass balance between glucose utilization and NADPH demand for oxidative burst: over the 1-h period following PMA stimulation, neutrophils (per 10,000 cells) needed to produce at least 0.84 ± 0.12 nmol NADPH from oxPPP to power up the oxidative burst shown in Fig. 1d. This was quantified based on the difference in area under the curve of oxygen consumption in PMA-stimulated control versus the oxidative burst curve when oxPPP was inhibited by G6PDi, and the biochemical knowledge that at least one NADPH is needed to reduce every two molecules of oxygen. Over this 1-h period, cells consumed a total of 0.28 ± 0.05 nmol of glucose as measured by the decrease in glucose content in spent media. The ratio of total NADPH production from oxPPP to total glucose consumption was greater than 2, which is the maximal expected NADPH yield when 100% of consumed glucose is shunted into oxPPP. This observation poses an important question—how do neutrophils produce NADPH at such high rate to enable substantial oxidative burst?

**Extended Data Fig. 2:**
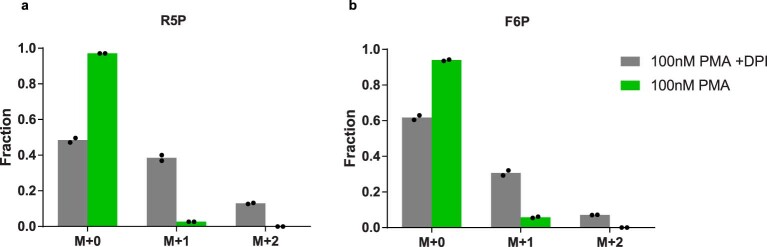
Labeling patterns of ribose-5-phosphate (a) and fructose-6-phosphate (b) from 1-^13^C-glucose (Related to Fig. 2). Labeling patterns were measured in neutrophils stimulated with PMA (100 nM) for 30 min, with or without the treatment of NOX inhibitor DPI (10 µM). Bar graphs show the mean value from two technical replicates (indicated by dots). Results have been confirmed in three independent experiments. Labeling patterns of selected other metabolites are shown in Fig. 2. *F6P peak may contain some glucose-1-phosphate, as these two compounds are not fully resolved on LCMS. Source data

One way to achieve high NADPH yield from limited glucose is to recycle pentose carbon via non-OxPPP and reversed upper glycolysis^44^. This will allow each molecule of glucose to be oxidized via oxPPP multiple times. Labelling results from 1-^13^C-glucose tracing suggest that such a metabolic mode may occur in activated neutrophils. Without oxidative burst, pentose phosphates were substantially labelled from 1-^13^C-glucose while during oxidative burst this labelling was almost completely lost (Fig. 2d and Extended Data Fig. 2a), indicating that oxPPP becomes the main source of pentose phosphate production. Unlabelled pentose phosphates overflowing from oxPPP can be converted by non-OxPPP to the glycolic intermediates F6P and glyceraldehyde 3-phosphate (GAP). Consistent with PPP-driven F6P production, we found that F6P labelling also substantially decreased during oxidative burst (Extended Data Fig. 2b). Very intriguingly, this great loss of labelling was also observed in G6P (Fig. 2e), indicating that unlabelled PPP-derived F6P is recycled back to G6P via reversed GPI flux.

**Fig. 3:**
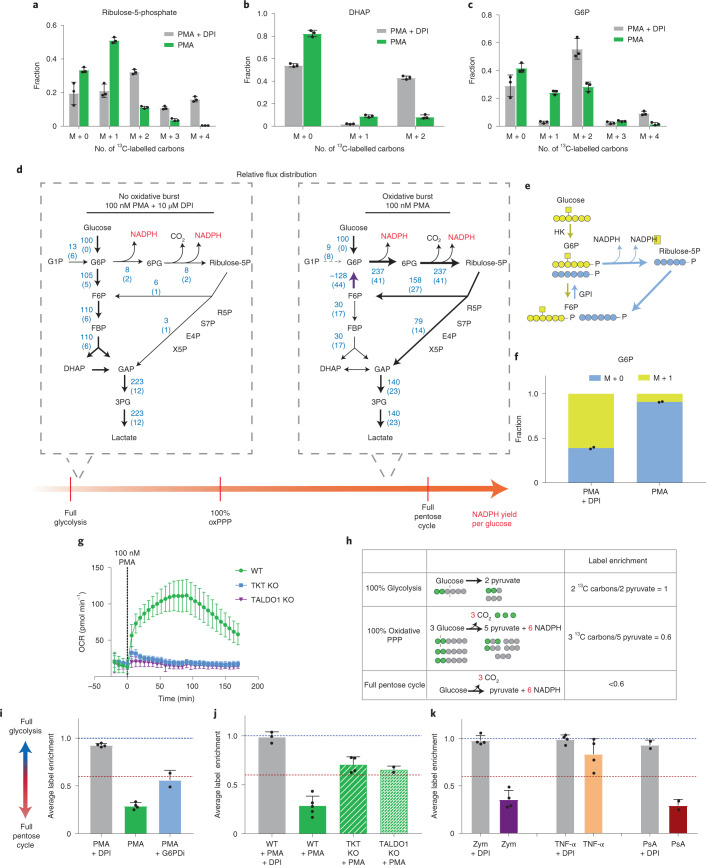
Neutrophils adopt pentose cycle during oxidative burst. **a**–**c**, Labelling patterns of ribulose-5-phosphate (**a**), DHAP (**b**) and G6P (**c**) from 1,2-^13^C-glucose tracing. Bar graphs represent mean ± s.d., *n* = 3 biological replicates (neutrophils isolated from different donors represented by individual dots). **d**, Metabolic flux distributions in stimulated neutrophils with or without oxidative burst. Numbers in blue next to the reaction indicate relative net flux, which are normalized to HK flux as 100. Values represent average results from multiple donors, with s.d. indicated in parentheses (*n* = 3 for no oxidative burst condition, *n* = 5 for oxidative burst condition). **e**, Schematic showing G6P labelling from 3-^2^H-glucose. HK activity generates ^2^H-labelled G6P, whereas pentose cycle generates unlabelled G6P. **f**, Labelling pattern for G6P from 3-^2^H-glucose. Bar graph represents mean value of *n* = 2 technical replicates indicated by individual dots. Labelling in PMA-stimulated oxidative burst was repeated in four independent experiments. **g**, PMA-induced oxidative burst is suppressed in TKT or TALDO1 KO HL-60 cells (mean ± s.d., *n* = 6 (WT) and *n* = 12 (TKT and TALDO1 KO) independent samples). **h**, Schematic illustrating expected average labelling enrichment from 1,2-^13^C-glucose when cells metabolize glucose: (1) 100% via glycolysis, (2) 100% via oxPPP with no recycling (no GPI flux) or (3) via full pentose cycle with reversed GPI net flux (that is, oxPPP flux is 300% of total glucose uptake). **i**, Average labelling enrichment of DHAP from 1,2-^13^C-glucose in human peripheral blood neutrophils stimulated with PMA (100 nM) with or without NOX inhibitor DPI (10 µM) or G6PDi (50 μM). Mean ± s.d., dots represent results in different biological replicates (neutrophils from different donors), *n* = 4 (PMA, PMA + DPI), *n* = 2 (PMA + G6PDi). **j**, Average labelling enrichment of DHAP from 1,2-^13^C-glucose from WT, TKT or TALDO1 KO HL-60 cells. Mean ± s.d. from independent samples measured in independent experiments; *n* = 2–5 in each specific condition, as indicated by individual dots. **k**, Average labelling enrichment of DHAP from 1,2-^13^C-glucose in human peripheral blood neutrophils stimulated ex vivo with zymosan A (300 μg ml^–1^), TNF-α (100 ng ml^–1^) or PsA (10:1 bacteria/neutrophils) with or without DPI (10 µM). Mean ± s.d., dots represent different biological replicates (different donors), *n* = 4 (zym, TNF-α), *n* = 2 (PsA). Source data

**Extended Data Fig. 3:**
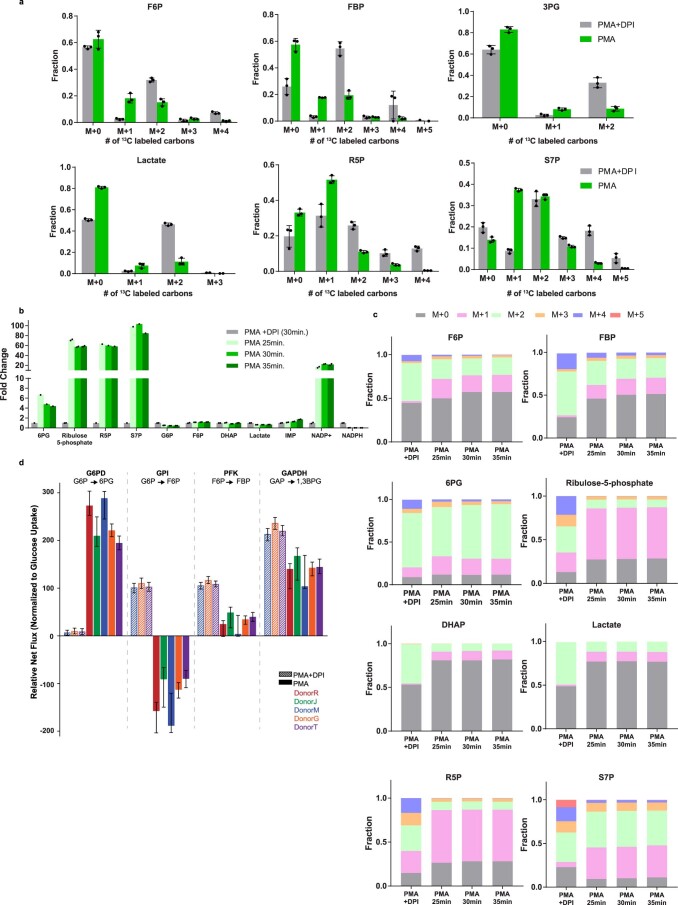
1,2-^13^C glucose tracing and metabolic flux analysis indicate neutrophils switch to pentose cycle during oxidative burst induced by PMA (Related to Fig. 3). a. Labeling pattern of selected PPP and glycolytic intermediates from 1,2-^13^C glucose in neutrophils stimulated with PMA (100 nM) for 30 min, with or without the treatment of DPI (10 µM). Mean ± SD, N = 3 biological replicates. Dots indicate results measured in neutrophils from each individual donor. *F6P peak may contain some glucose-1-phosphate, as these two compounds are not fully resolved on LCMS. b,c. Glycolysis and PPP reach pseudo-steady state at the peak of oxidative burst (~30 min post stimulation), as indicated by steady metabolite abundances (b) and labeling patterns (c) 25, 30, 35 min post- PMA stimulation. Bar graph show mean value of technical replicates when applicable. Trends have been repeated in two independent experiments. d. Relative net flux of key reactions determined in neutrophils isolated from different donors (indicated by different colors). Hatched bars are the fluxes in absence of oxidative burst (30 min after stimulation with 100 nM PMA, with 10 μM DPI treatment) and solid bars are the fluxes during oxidative burst (30 min after stimulation with 100 nM PMA). Error bars indicate the confidence intervals from each flux analysis. Source data

To further probe recycling, we performed tracing experiments with the 1,2-^13^C-glucose tracer. OxPPP activity will uniquely separate glucose C1 from C2, resulting in downstream metabolites being singly labelled, whereas scrambling by non-OxPPP will not^45^. During oxidative burst there is a large increase in the 1-labelled fraction of pentose phosphates, and over 20-fold increase in the M + 1/M + 2 ratio of lower glycolytic compounds such as DHAP (Fig. 3a,b and Extended Data Fig. 3a), confirming a drastic increase in oxPPP activity. Without oxidative burst, G6P is mainly 2-labelled, as expected from direct hexose kinase (HK) activity, with a notable fraction being 4-labelled, which indicates that non-OxPPP and GPI are highly reversible at baseline. Importantly, during oxidative burst we observed the appearance of substantial 1-labelled G6P, which indicates that glucose carbon that had gone through oxPPP is recycled back to G6P (Fig. 3c).

To quantitively characterize the rewiring of glucose metabolism coupled to oxidative burst, we performed metabolic flux analysis in neutrophils during oxidative burst (30 min post-PMA stimulation) or without oxidative burst (also stimulated with PMA for 30 min, but treated with DPI). The 1,2-^13^C-glucose tracing data were fitted using the isotopomer network compartmental analysis (INCA) platform^46^ (Supplementary Table 1) under a pseudo-steady-state framework, because although PMA stimulation induces very rapid metabolic changes, around the peak of oxidative burst (~30 min after stimulation) the system has reached a new steady state, as indicated by steady oxygen consumption (Fig. 1d), metabolite levels and labelling patterns (Extended Data Fig. 3b,c). This analysis revealed drastic changes in flux distribution consistent across donors (Fig. 3d, Extended Data Fig. 3d and Supplementary Table 2): with the activation of oxidative burst, oxPPP flux increased from <10% to >200% of glucose uptake rate and glycolysis became uncoupled. Net flux through upper glycolytic reaction GPI flipped from close to 100% of glucose uptake towards F6P to more than 100% reversed, while lower glycolysis (GAP to lactate) became mainly supplied by PPP-derived GAP. This drastic switch from glycolysis to cyclic PPP demonstrates that neutrophils have remarkable metabolic flexibility that allows them to produce NADPH at an ultra-high yield to fuel oxidative burst.

To further verify carbon recycling during oxidative burst, we applied 3-^2^H-glucose tracing. This tracer can be directly converted to ^2^H-labelled G6P by HK. However, if cells engage in pentose cycle, deuterium at the C3 position is transferred to NADPH when G6P enters oxPPP for the first time, and therefore G6P generated from recycling via reversed GPI is unlabelled (Fig. 3e). We found that, without oxidative burst, the majority of G6P is deuterium labelled, while during oxidative burst it is mostly unlabelled (Fig. 3f), indicating that cells adopt a substantial pentose cycle.

**Extended Data Fig. 4:**
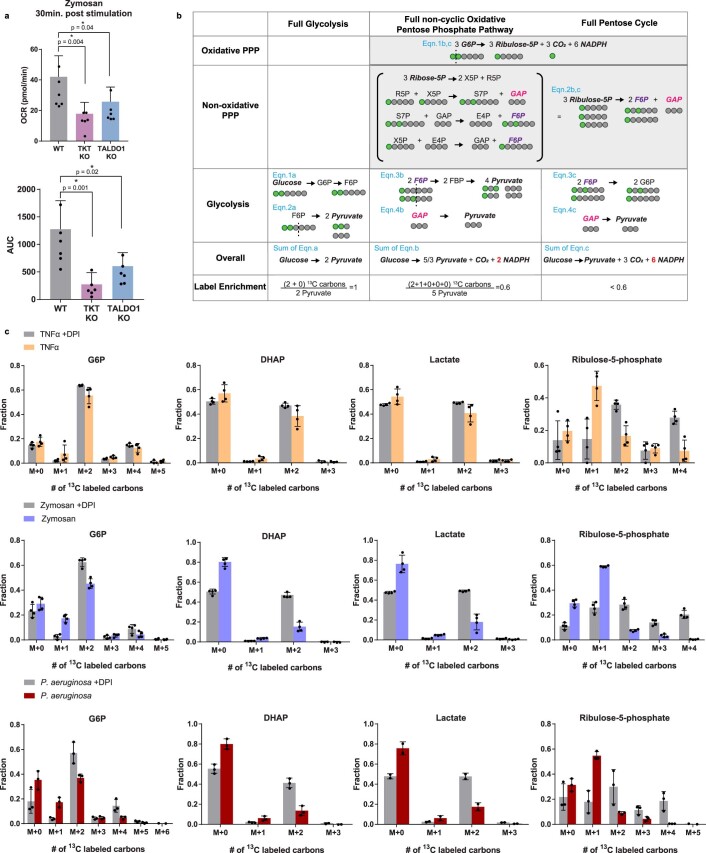
Metabolic shift towards pentose cycle during oxidative burst (Related to Fig. 3). a. Zymosan-induced oxidative burst depends on non-oxidative PPP activity. The oxygen consumption rate at the peak of zymosan-induced burst (30 min post-stimulation, upper panel), and the area under curve over 1 h of oxidative burst after zymosan stimulation (lower panel) in wild-type, TKT knockout and TALDO1 knockout HL-60 cells. Mean ± SD, N = 6 independent samples. P-values are determined by two-sided, unpaired t-test. b. Average labeling enrichment in lower glycolytic products (for example pyruvate and lactate) from 1,2-^13^C-glucose indicates the general flux distribution of glycolysis and PPP. Metabolite labeling from 1,2-^13^C-glucose under 3 extreme metabolic modes was laid out here: (i) Full glycolysis: When 100% of the glucose is metabolized via glycolysis with no oxidative PPP activity; (ii) Full non-cyclic oxidative PPP: oxidative PPP flux is 100% of glucose uptake rate, one labelled carbon per glucose molecule is released as CO_2_. There is no conversion between G6P and F6P. (iii) Full pentose cycle: All G6P is metabolized via the oxidative PPP. Additionally, all F6P produced from the non-oxidative PPP is recycled back to G6P via reversed GPI activity. c. Labeling patterns of key PPP and glycolytic intermediates from 1,2-^13^C- glucose during oxidative burst induced by various stimuli, compared to corresponding no oxidative burst conditions (stimulated with the same stimulus, treated with 10 µM DPI). To induce oxidative burst, human peripheral blood neutrophils were stimulated with Zymosan A (300 μg/mL), TNFα (100 ng/mL) or Pseudomonas aeruginosa (10:1 PsA:neutrophil) for 30 min. Plot show mean ± SD from biological replicates. Individual dots represent results in neutrophils from different donors N = 4 (Zymosan, TNFα) N = 3 (PsA). Source data

To experimentally test the significance of pentose cycling, we knocked out either transketolase (TKT) or transaldolase (TALDO1), two enzymes in non-OxPPP required for recycling but that do not directly produce NADPH, in neutrophil-like cell line HL-60. Wild-type (WT) HL-60 cells displayed a strong oxidative burst when stimulated with PMA, but this capability was largely lost in *TKT* and *TALDO1* KO cells (Fig. 3g). Similarly, we found that *TKT* or *TALDO1* KO also significantly suppressed zymosan-induced oxidative burst (Extended Data Fig. 4a). These results demonstrate that pentose cycle is required for oxidative burst.

### Quantitative coupling between pentose cycle and oxidative burst

Without performing modelling-based metabolic flux analysis, the general glucose metabolism mode can be indicated by average labelling enrichment (defined by the average number of ^13^C-labelled carbons per molecule) in lower glycolytic compounds such as DHAP and lactate from 1,2-^13^C glucose tracing. Given that in stimulated neutrophils glucose is the main source of glycolysis and PPP, we consider three extreme glucose metabolism modes (Fig. 3h and Extended Data Fig. 4b): When all glucose is metabolized via glycolysis, each molecule of 1,2-^13^C glucose yields one unlabelled pyruvate and one 2-labelled pyruvate, resulting in average labelling enrichment of 2/2 = 1. As cells engage in more oxPPP, more labelled carbon from C1 position would be released as CO_2_. When 100% of glucose is shunted into oxPPP but cells do not engage in net cycling, from every molecule of 1,2-^13^C glucose up to one labelled carbon is lost as ^13^CO_2_ and the reaction yields 5/3 pyruvate per glucose, resulting in the lower limit of average labelling enrichment in pyruvate of 0.6. As cells further engage in recycling with reversed GPI, more labelled carbon can be released as ^13^CO_2_ when glucose enters oxPPP for the second or third round, thus resulting in labelling enrichment in lower glycolysis <0.6. In human peripheral blood neutrophils, labelling enrichment was close to 1 without oxidative burst but dropped to <0.3 during PMA-induced oxidative burst, confirming the shift from glycolysis-dominant metabolism to pentose cycle. Partial inhibition of oxPPP with G6PDi significantly increased labelling enrichment, as expected (Fig. 3i). Similarly, in HL-60 cells, PMA-stimulated oxidative burst caused a decrease in labelling enrichment, from ~1.0 to ~0.3, and KO of non-OxPPP enzymes TKT or TALDO1 significantly increased labelling enrichment to slightly >0.6, which corresponds to near full oxPPP but with no cycling, as expected (Fig. 3j).

Using this approach, we then examined metabolic shift coupled to the oxidative burst induced by zymosan, PsA and TNF-α. Like PMA, all three stimuli induced a shift towards pentose cycle as indicated by a decrease in lower glycolysis labelling enrichment (Fig. 3k) and increase in M + 1 fraction in pentose phosphate and G6P (Extended Data Fig. 4c). Zymosan and PsA induced a large shift to net cycling (labelling enrichment <0.6), while TNF-α caused only a small shift towards higher oxPPP without engaging in net cycling. Across these stimuli, the extents of metabolic shift correlate with the strength of oxidative burst (Fig. 1b) included by them.

**Fig. 4:**
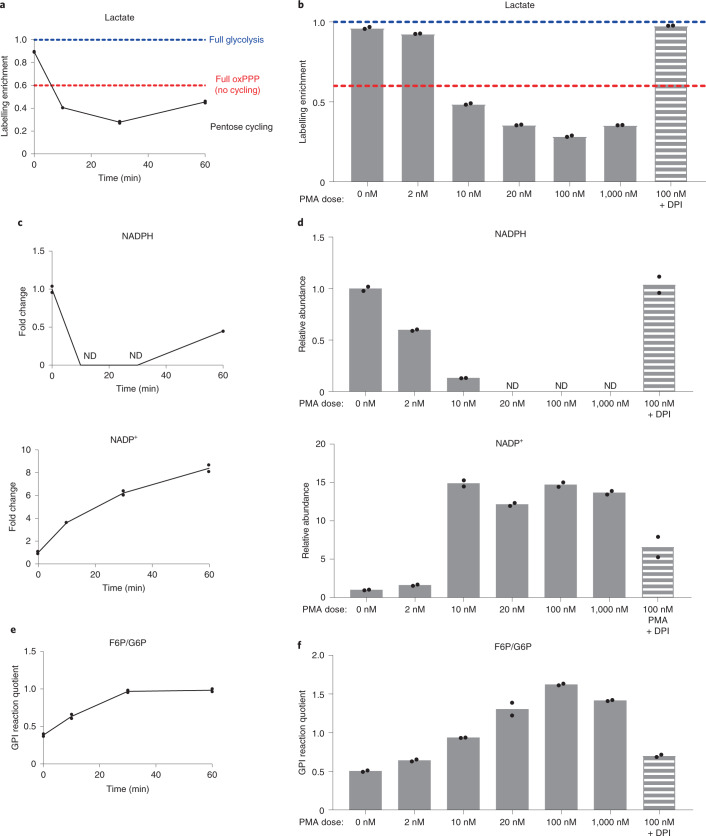
Changes in metabolic state over time and with varying doses of PMA. **a**,**b**, Average labelling enrichment of lactate from 1,2-^13^C-glucose changes over time following stimulation with 100 nM PMA (**a**), and with varying doses of PMA (at 30 min) or with the treatment of 10 μM DPI in addition to PMA stimulation (**b**). **c**,**d**, Relative abundances of NADPH (top) and NADP^+^ (bottom) change over time after stimulation with 100 nM PMA (**c**), and with varying doses of PMA (at 30 min) (**d**). **e**,**f**, Reaction quotient of GPI—that is, the ratio between F6P and G6P concentrations—changes over time after simulation with 100 nM PMA (**e**) and with increasing doses of PMA or with the treatment of 10 μM DPI in addition to PMA stimulation (**f**). Graphs show mean value of two technical replicates, represented by individual dots. All trends were repeated in at least two independent experiments. Source data

**Extended Data Fig. 5:**
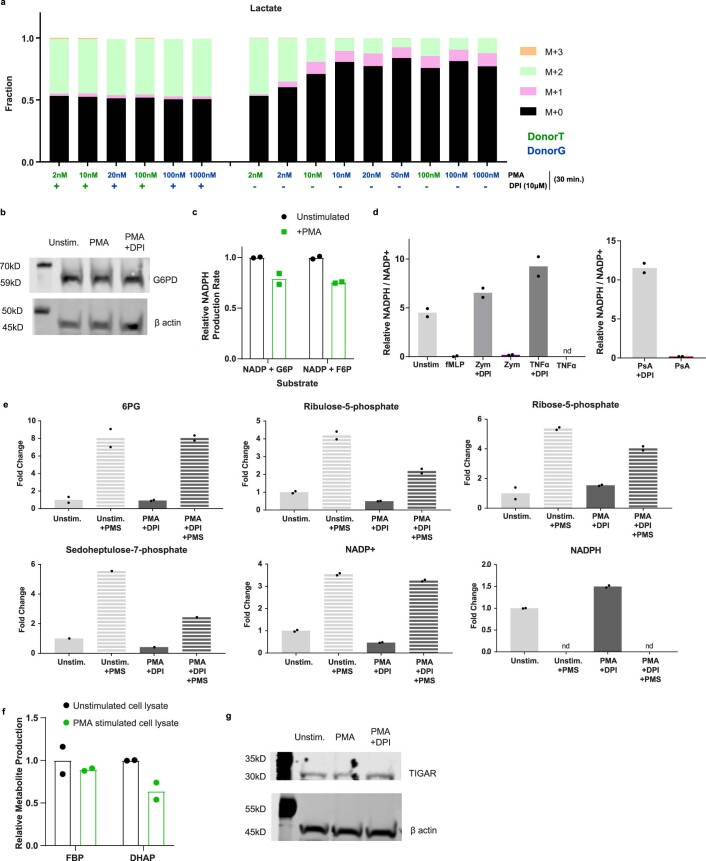
Regulatory mechanisms for the rapid shift towards pentose cycle (Related to Fig. 4). a. Lactate labeling from 1,2-^13^C glucose after 30 minutes stimulation with various doses of PMA, with or without NOX inhibitor DPI (10 µM). Due to limited neutrophil yield from each blood draw, the entire span of dose-response curve was covered from two different donors (indicated by color code), with several overlapping conditions to ensure the trend and main observations are consistent across donors. Bar graphs present the mean value of technical duplicates. b. G6PD protein level in unstimulated, PMA (100 nM, 30 min)-stimulated, and PMA (100 nM) + DPI (10 μM) treated HL-60 cells. Results confirmed in two independent experiments (see source data). c. Relative NADPH producing rate measured in cell lysates of unstimulated and PMA (100 nM)- stimulated HL-60 cells, when cells were given G6P or F6P as substrate. Relative rate is normalized to the NADPH production in unstimulated cell lysate with G6P as substrate. Results have been repeated in two independent experiments. d. Relative redox ratio of NADPH/NADP^+^ is depleted during oxidative burst induced by various stimuli. NADPH and NADP^+^ were measured in human blood neutrophils stimulated with fMLP (100 nM) for 10 min, or Zymosan A (300 μg/mL), TNFα (100 ng/mL), or *Pseudomonas aeruginosa* (10:1 PsA:neutrophil) for 30 min. Nd. Indicates NADPH was not detectable. e. Treatment of electron accepter Phenazine Methosulfate (PMS) (10 μM) causes accumulation of PPP intermediates, increase in NADP + , and depletion of NADPH in neutrophils that do not engage in oxidative burst. These changes were seen either in unstimulated human neutrophils or PMA (100 nM)-stimulated neutrophils treated with 10 μM DPI to eliminate oxidative burst. f. PFK and aldolase enzyme activity measured in cell lysates of unstimulated- and PMA (100 nM) stimulated- HL-60 cells. Lysates were given substrates F6P and ATP, and the production rate of FBP and DHAP were measured and normalized to unstimulated condition. c-f. Bar graph present mean value of N = 2 technical replicates (indicated by dots) g. The level of TIGAR in HL-60 cells that are unstimulated, stimulated with 100 nM PMA for 30 min, or stimulated with 100 nM PMA and treated with DPI. Results have been confirmed in two independent experiments (see source data). Source data

We observed similar quantitative coupling between the metabolic shift toward the pentose cycle and the strength of oxidative burst over both time and with different stimulation dosages. Upon stimulation with 100 nM PMA, labelling enrichment in lactate dropped from ~1.0 to well below 0.6 as soon as 10 min, further decreased to a minimum at 30 min when oxidative burst peaked, then rose to around 0.5 at 60 min when oxidative burst dampened (Fig. 4a). This temporal correlation suggests that neutrophils promptly adjust metabolic strategy to supply NADPH according to changing demands during oxidative burst. Over increasing dosages of PMA, labelling enrichment decreased in a dose-dependent manner and reached a minimum at ~100 nM, where peak oxidative burst reached maximum (Fig. 4b). Elimination of oxidative burst by DPI brought labelling enrichment back to near 1.0 at any dose of PMA (Extended Data Fig. 5a), suggesting that, without oxidative burst, stimulated neutrophils use mainly glycolysis. Interestingly, our flux analysis showed that neutrophils at maximal oxidative burst (100 nM PMA) use nearly the complete pentose cycle (indicated by PFK net flux approaching zero in some donors (Extended Data Fig. 3d)), and thus approach the upper limit of NADPH yield from glucose (without engaging substantial activity of the gluconeogenesis enzyme FBPase). The fact that, over increasing dosages of PMA or zymosan, cells eventually reach a common maximal oxidative burst magnitude (Fig. 1i and Extended Data Fig. 1d) probably reflects that maximal metabolic capacity to produce NADPH defines the maximal magnitude of oxidative burst.

### Mechanisms for rapid switching towards pentose cycle

This study reveals the impressive metabolic flexibility of neutrophils. We next sought to understand the mechanism allowing neutrophils to rapidly switch to pentose cycle on demand. The best-known rate-limiting step of oxPPP is G6PD. We found that neither G6PD protein levels nor maximal enzymatic activity (as measured in cell lysate) increased during oxidative burst (Extended Data Fig. 5b,c). This is consistent with the observation that the profound oxPPP upregulation can occur within minutes, a time frame faster than most transcriptional or translational regulation mechanisms can achieve, pointing to plausible small-molecule-driven regulation.

OxPPP uses NADP^+^ as a substrate, and NADPH is a known feedback inhibitor. It has been suggested that G6PD runs at a very low percentage of its maximal capacity in many cells due to a low cellular NADP^+^/NADPH ratio, which gives cells a large reserved oxPPP capacity and allows G6PD flux to be very sensitive to NADP^+^/NADPH redox ratio, which often indicates NADPH demand^22,47,48^. As a result, an increased NADP^+^/NADPH ratio has been shown to effectively drive the activation of oxPPP flux from *Escherichia coli* to human cells^21,22,32,49^. Upon neutrophil activation, NOX can quickly consume NADPH and oxidize it to NADP^+^^4,50^. We expect that NOX activation, together with activation of NAD kinase^51^, can cause accumulation of NADP^+^ and depletion of NADPH, and thus increase oxPPP flux without increased G6PD enzyme activity. Indeed, we observed strong temporal- and dose-dependent correlation between the decrease in NADPH/NADP^+^ ratio and increase in oxPPP (Fig. 4c,d). Upon stimulation with 100 nM PMA, NADPH dropped to a non-detectable level as soon as 10 min, stayed minimal at 30 min and slightly increased by 60 min when the oxidative burst had dampened, while NADP^+^ continued to accumulate over time (Fig. 4c). With increasing doses of PMA, NADPH depletes and NADP^+^ accumulates, reaching a maximum at ~20 nM when oxidative burst and the upregulation of oxPPP approach maximum (Fig. 4d). Similar depletion of the NADPH/NADP^+^ ratio was also observed with stimulation by fMLP, zymosan, TNA-α and PsA (Extended Data Fig. 5d). Inhibition of NOX fully prevented the depletion of NADPH and substantially reduced accumulation of NADP^+^ (Fig. 4d). These results suggest that activation of NOX dynamically controls the cellular NADPH/NADP^+^ ratio, which then results in rapid upregulation of oxPPP flux. Consistent with this mechanism, treatment of neutrophils without oxidative burst (either unstimulated neutrophils or PMA-stimulated neutrophils treated with NOX inhibitor) with an electron sink phenazine methosulfate^52^ to deplete NADPH induces substantial upregulation of PPP metabolites, which mimics that observed during oxidative burst (Extended Data Fig. 5e).

Apart from drastic oxPPP upregulation, remodelling of glycolysis is also associated with pentose cycle. It has been reported that blockage of glycolysis can help cells divert more flux into oxPPP under oxidative stress^23,33,53^, and TIGAR-driven inhibition of PFK is one known mechanism of glycolysis inhibition^54^. We therefore measured the activities of PFK and aldolase in cell lysates and found that, during PMA-induced oxidative burst, neither TIGAR levels nor F6P-driven FBP production changed significantly while DHAP production reduced slightly (Extended Data Fig. 5f,g). Another report showed that treatment of cells with a PFK activator significantly lowered oxidative burst^40^. Activation of PFK can drain F6P. In the present study we found that the ratio between F6P and G6P is particularly important in controlling the reversal of GPI net flux, which is required for substantial pentose cycling. Our flux analysis revealed large back-and-forth GPI gross flux even at baseline without oxidative burst (Supplementary Table 2), suggesting that GPI rests at a near-equilibrium state in neutrophils and consistent with the knowledge biochemically that GPI reaction has a very small ΔG^0^ (change in Gibbs free energy)^55^. Therefore, thermodynamically, GPI net flux can be readily flipped by changes in its reaction quotient—that is, the ratio between F6P and G6P concentrations. Indeed, during oxidative burst the [F6P]/[G6P] ratio increases in a time- and dose-dependent manner, closely correlating with the flip of GPI net flux (Fig. 4e,f). This increase is probably due to both high oxPPP activity during oxidative burst that rapidly consumes G6P, lowering its level (Extended Data Fig. 3b), and increased non-OxPPP flux that produces F6P, while potential inhibition of glycolysis downstream of F6P may also contribute. Consistent with the fact that GPI is highly reversible and not rate limiting in pentose cycle, we found that using F6P as substrate in cell lysate can drive NADPH production at a very similar rate to using G6P as substrate (Extended Data Fig. 5c). Together, these results suggest that near-equilibrium upper glycolysis and reserved capacity in oxPPP enables the remarkable metabolic flexibility found in neutrophils.

### Pentose cycle is required for neutrophil functions

**Fig. 5:**
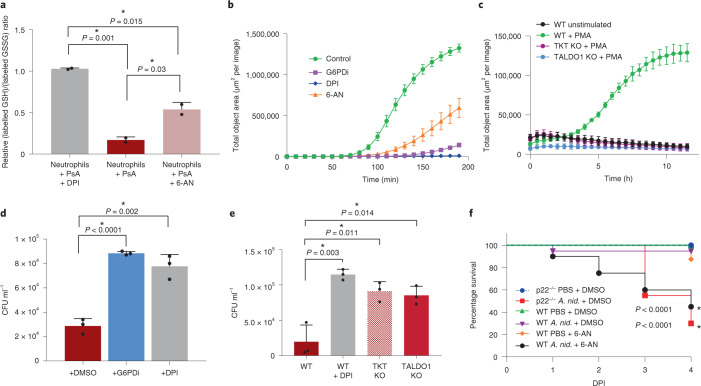
Disruptions of pentose cycle limit neutrophil functions and pathogen killing. **a**, Relative ratio of reduced to oxidized glutathione in PsA internalized by human peripheral blood neutrophils. Bar graph shows mean ± s.d. from *n* = 2 independent experiments using neutrophils from different donors (represented by individual dots). *P* values were determined by two-tailed, unpaired *t*-test. **b**, NET release by human peripheral blood neutrophils induced by PMA (100 nM) is suppressed by treatment with G6PDi (50 μM), 6-AN (5 mM) or DPI (10 μM). **c**, NET release by HL-60 cells induced by PMA stimulation (100 nM) is suppressed by TKT or TALDO1 KO. **b**,**c**, Plots show mean ± s.d. from one representative experiment with *n* = 6 technical replicates. Results were confirmed in three independent experiments with human peripheral blood neutrophils and in two independent experiments with HL-60 cells. **d**, Residual CFU of PsA following incubation with human peripheral blood neutrophils treated with G6PDi (50 μM), DPI (10 μM) or vehicle control (DMSO). **e**, Residual CFU of PsA following incubation with WT HL-60 cells with or without treatment by DPI (5 μM), or with TKT or TALDO1 KO HL-60 cells. **d**,**e**, Plots show mean ± s.d. from one representative experiment with *n* = 3 technical replicates. Results were confirmed in at least two independent experiments. *P* values were determined by two-tailed, unpaired *t*-test. **f**, Survival curve of zebrafish following infection with *A. nidulans* (*A. nid*.) is impacted by PPP activity and oxidative burst. WT zebrafish, or zebrafish with KO of the p22 subunit of NOX, were challenged with *A. nidulans* or PBS control. To test the impact of PPP, WT fish were treated with either 6-AN (1 mM) or vehicle control (DMSO); *n* = 17 (p22^–/–^ PBS + DMSO); *n* = 20 (p22^–/–^ *A. nid.* + DMSO); *n* = 22 (WT PBS + DMSO); *n* = 19 (WT *A. nid.* + DMSO); *n* = 24 (WT PBS + 6-AN); *n* = 20 (WT *A. nid*. + 6-AN) *P values were determined by log-rank (Mantel–Cox) test. Source data

Neutrophils produce a large amount of ROS from oxidative burst to kill pathogens, partly by causing oxidative stress^56^. We coincubated ^13^C-labelled PsA with human neutrophils and found the bacterial glutathione/glutathione disulfide (GSH/GSSG) ratio greatly reduced by neutrophil oxidative burst (Fig. 5a), as expected. Treatment of neutrophils with oxPPP inhibitor significantly alleviated oxidative stress in bacteria, as indicated by lower reduction of bacterial GSH/GSSG (Fig. 5a). It is worth noting that, because bacteria can also use oxPPP to defend against oxidative stress, if the inhibitor also affects bacterial oxPPP, it would reduce the bacterial GSH/GSSG ratio even further. Therefore, this restoration of bacterial GSH/GSSG ratio shows that PPP activity in neutrophils is critical in the induction of oxidative stress in phagocytized bacteria.

Besides acting directly as pathogen-killing agents, ROS can also induce the release of NET^3^, which starts shortly after oxidative burst. We found that either inhibition of oxPPP or KO of non-OxPPP enzymes TKT or TALDO1 greatly suppressed NET release, similar to direct inhibition of NOX (Fig. 5b,c and Supplementary Fig. 1), demonstrating the importance of pentose cycle in NET release.

Finally, to assess the overall impact of pentose cycle on pathogen killing, we coincubated PsA with neutrophils. Both inhibition of oxPPP and KO of non-OxPPP enzymes greatly suppressed pathogen-killing capacity in vitro (as indicated by significantly higher remaining bacterial colony-forming units (CFU) after coincubation), to an extent similar to direct inhibition of oxidative burst (Fig. 5d,e). To assess defence against pathogens in vivo, we used a fungal infection model in zebrafish. It was previously established that oxidative burst by neutrophils is critical for the clearance of *Aspergillus nidulans* infection^57^. When challenging zebrafish with *A. nidulans*, we found that PPP inhibition greatly reduced host survival, similar to the genetic mutation of a NOX complex component (*p22*^–/–^) that disrupts oxidative burst (Fig. 5f). Together, these data demonstrated the critical role of PPP in defence against pathogens.

### Shift towards PPP during other innate immune responses

**Fig. 6:**
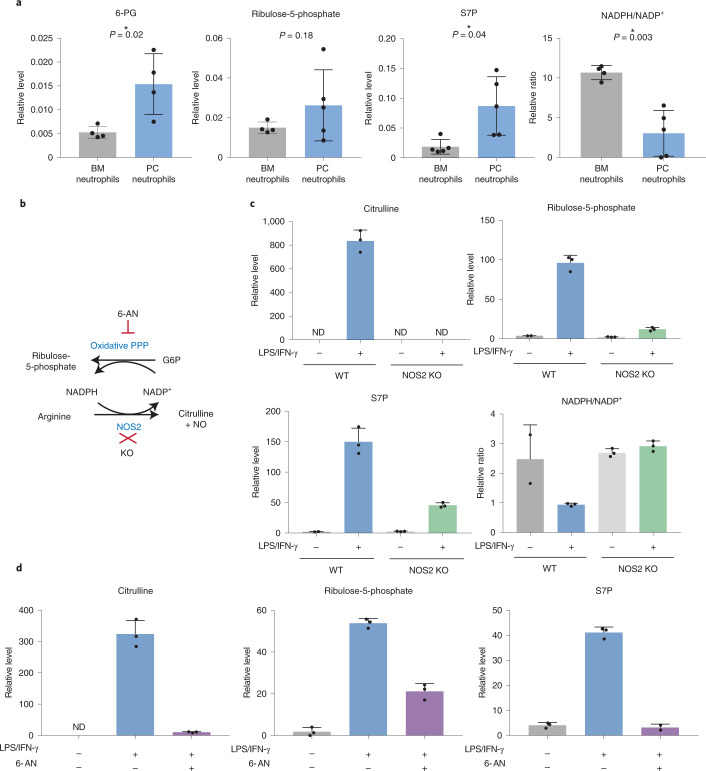
Rewiring of PPP and changes in redox status in murine neutrophils activated in vivo and in classically activated macrophages. **a**, Relative NADPH/NADP^+^ redox ratio and PPP metabolite levels in neutrophils isolated from either bone marrow (BM) or peritoneal cavity (PC) of mice following peritoneal injection of MSU. Bar graphs show mean ± s.d., dots represent biological replicates; *n* = 5 mice. Numbers of effective measurements of specific compounds per condition (indicated by individual dots) vary due to insufficient number of neutrophils isolated from bone marrow in one of the mice for the complete LC–MS analyses. *P* values were determined by two-sided, unpaired *t*-test. **b**, Schematic showing the connection between PPP and NOS2 in macrophages. **c**, Metabolite abundance in WT or *NOS2* KO bone marrow-derived macrophages stimulated with LPS and IFN-ɣ. Mean ± s.d., *n* = 3 Technical replicates. Results were repeated in two independent experiments. **d**, Metabolite abundance in WT bone marrow-derived macrophages stimulated with LPS and IFN-γ for 48 h with or without treatment by 6-AN (1 mM). Mean ± s.d., *n* = 3 technical replicates. Source data

To test whether the rewiring of PPP, which we consistently observed with several types of ex vivo stimulation, also occurs when neutrophils are activated in vivo, we analysed metabolites in a peritonitis model in mice^58^. Following peritoneal injection of monosodium urate (MSU), neutrophils were isolated both from peritoneal lavage, which mainly contained activated neutrophils, and the bone marrow, which mainly contained non-activated neutrophils. We found that, similar to human neutrophils activated ex vivo, in vivo activated neutrophils showed accumulation of PPP intermediates and depletion of the NADPH/NADP^+^ ratio (Fig. 6a).

The production of reactive oxygen and nitrogen species (RONS) using NADPH as the reducing power for pathogen killing is a common function of several types of innate immune cells. For instance, classically activated macrophages can produce a large amount of nitric oxide (NO) using inducible nitric oxide synthase (NOS2), an enzyme that uses a mechanism very similar to NOX (Fig. 6b)^59^. To evaluate the relevance of the metabolic shift toward PPP in RONS production beyond neutrophils, we activated bone marrow-derived macrophages with lipopolysaccharide/ interferon-γ (LPS/IFN-y), which induces NOS2. We found that activation caused not only great accumulation of the NOS2 product citrulline, but also profound accumulation of PPP intermediates and reduced NADPH/NADP^+^ ratio (Fig. 6c), very similar to that observed in activated neutrophils. These changes were prevented when NOS2 was knocked out (Fig. 6c). Conversely, inhibition of oxPPP not only dampened the accumulation of PPP intermediates but also greatly suppressed NOS2 activity, as indicated by the absence of citrulline production (Fig. 6d). These results demonstrate the interdependence between the shift towards PPP for NADPH supply and activation of RONS production in macrophages, very similar to the interdependence observed in neutrophils.

## Discussion

**Fig. 7:**
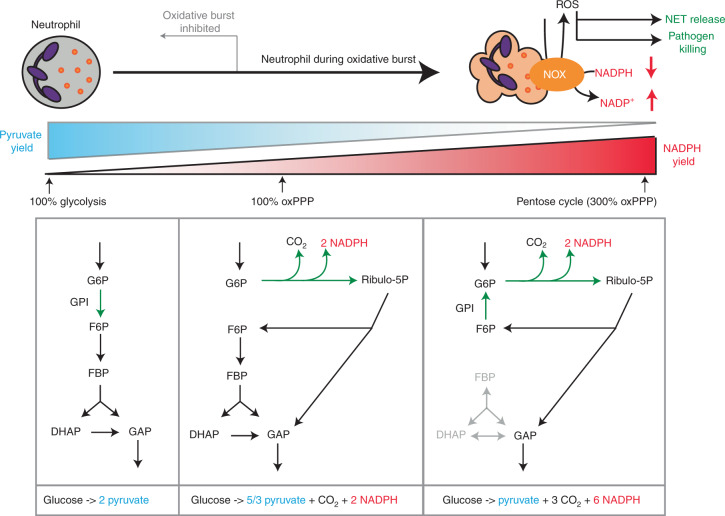
Reversed upper glycolysis and rapid activation of oxidative PPP supports oxidative burst in neutrophils. The model presents three different metabolic modes—glycolysis, full non-cyclic oxidative PPP and full pentose cycle—on a spectrum of NADPH yield. Quantitatively coupled to the activation of oxidative burst, neutrophils shift from a glycolysis-dominant metabolic mode with minor oxPPP to near-complete pentose cycle, to support NADPH demand and downstream function including pathogen clearance.

Cells can utilize nutrients through different metabolic routes to generate cofactors, energy and biosynthetic precursors at different proportions according to their needs. Specific to glucose, particular metabolic demands for ATP, NADPH, pyruvate and ribose are best matched with different metabolic modes^60^. Glycolysis produces the most energy, at the rate of two ATP and two pyruvate per glucose, but no NADPH. OxPPP produces two NADPH and one ribose, while partially oxidizing glucose. In cells where biosynthetic demand for ribose exceeds the need for NADPH, such as proliferating cancer cells, ribose can additionally be produced from non-OxPPP^61–63^. In cells with a greater demand for NADPH but not for ribose, like many non-proliferating cells, the overflowing ribose resulting from oxPPP can be shunted back to glycolysis as F6P and GAP via non-OxPPP. If the demand for NADPH further increases, some or all of the F6P can be recycled back to G6P for additional NADPH production, at the cost of higher carbon oxidation and lower ATP production. This forms the pentose cycle, which produces up to six NADPH, and one ATP and one pyruvate per glucose (Fig. 7). In mammalian cells oxPPP is usually a minor route for the metabolism of G6P, with flux <10% of glycolysis^64,65^. This is also the case for neutrophils when oxidative burst is not activated. Strikingly, within 30 min following stimulation, neutrophils shifted to near-complete pentose cycle and oxPPP flux rose to >twofold of glucose uptake. While pentose cycle has been long proposed in theory as an intriguing potential glucose metabolism mode with ultra-high NADPH yield, and some carbon recycling at gross flux level has been reported^21,22^, to our knowledge, substantial pentose cycling at net flux level (characterized by reversed GPI net flux, uncoupled glycolysis and oxPPP flux exceeding glucose uptake) has not been experimentally demonstrated in mammalian cells. This study shows that neutrophils have remarkable metabolic flexibility that enables them to produce NADPH rapidly and on demand.

**Extended Data Fig. 6:**
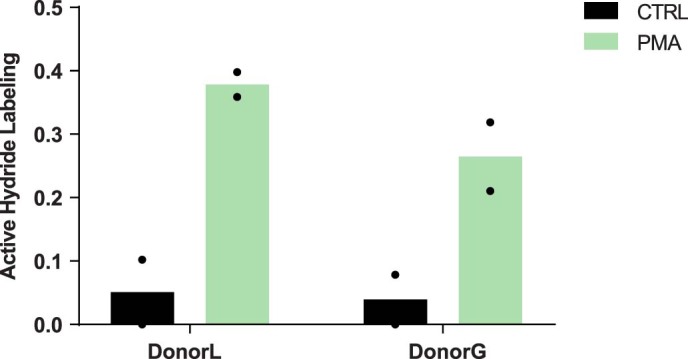
Majority of NADPH is generated from PPP during oxidative burst. Active hydride labeling of NADPH from 3-^2^H-glucose in unstimulated human peripheral blood neutrophils and neutrophils simulated by 100 nM PMA for 30 min. Labeling fraction at the active hydride was calculated by comparing the measured isotopic labeling pattern of NADPH to NADP^+^. Bar graph present mean value of N = 2 technical replicates (indicated by dots). Results have been confirmed in two independent experiments with neutrophils from two different donors, as indicated in the figure. Source data

Many pathways can potentially contribute to cellular NADPH production. Interestingly, using the 3-^2^H-glucose tracing approach^66^ we found that, in unstimulated neutrophils, the active hydride of NADPH barely labelled from 3-^2^H-glucose but the fraction of ^2^H-labelled NADPH increased drastically following PMA stimulation (Extended Data Fig. 6), suggesting that glucose-driven oxPPP becomes the dominant route for cellular NADPH production during oxidative burst even though it may not be the dominant source at baseline, which is consistent with the large increase in oxPPP flux measured by ^13^C labelling. This result suggests that neutrophils preferably rely on upregulation of oxPPP, rather than other pathways that can also contribute to NADPH production at baseline, to fulfil the large and rapid increase in NADPH demand associated with oxidative burst. This is probably because, in comparison to other pathways, PPP has greater reserve capacity^34,48^ and the fast-acting regulatory mechanism and near-equilibrium upper glycolysis allow cells to immediately elicit reserved NADPH-producing capacity when NOX activity alters redox ratio.

This study demonstrates that rapid shift to pentose cycle is essential for powering up oxidative burst and related effector functions in activated neutrophils, ultimately allowing them to promptly mount a first line of defence against pathogens. Consistently, patients with deficiencies in either the oxPPP enzyme G6PD or the non-OxPPP enzyme TALDO are found to be susceptible to recurrent infections and sepsis^67,68^. The reconfiguration of PPP is an impressive example of neutrophil metabolic flexibility, but not the only one. We have observed other rapid metabolic alterations following each of the stimulations (Fig. 1a). Some of these changes, including increase in glycolytic intermediates, are largely independent of oxidative burst (Fig. 1g). The accumulation in glycolytic intermediates is probably caused by the stimulation-induced increase in glucose transporters on the cell membrane, which increases glucose uptake rate^69,70^. This would further increase the absolute flux supply of oxPPP (in addition to the greatly increased relative flux distribution into oxPPP). Continued studies on metabolic rewiring in a wide range of pathways in neutrophils will reveal the biochemical basis of important responses in infection and inflammation, and guide the design of metabolic interventions to modulate innate immunity.

## Methods

### Isolation, culture and stimulation of human peripheral blood neutrophils

Human neutrophils were isolated from peripheral blood freshly collected from healthy donors, following the protocol approved by the University of Wisconsin Institutional Review Board (protocol no. 2019-1031-CP001). Informed consent was obtained from all participants. Neutrophils were purified using the MACSxpress Whole Blood Neutrophil Isolation Kit (Miltenyi Biotec, no. 130-104-434) followed by erythrocyte depletion (Miltenyi Biotec, no. 130-098-196) according to the manufacturer’s instructions. Neutrophils were spun down at 300*g* for 5 min, resuspended in RPMI 1640 (VWR, no. VWRL0106-0500) supplemented with 5 mM L-glutamine (Fisher Scientific, no. BP379-100) and 0.1% Human Serum Albumin (Lee Biosolutions, no. 101-15) and kept at 37^o^C in incubators under 5% CO_2_. Purity of isolated cells was verified by flow cytometry using antibodies against neutrophil surface markers CD11b (PE) (Biolegend, no. 301305) and CD15 (AlexaFluor700) (Biolegend, no. 301919), and cell viability was checked using Ghost Dye 450 (Tonbo Biosciences, no. 13-0863). Data were analysed with FlowJo software. Typically, isolated cells are >90% CD11b^+^ CD15^+^ and >95% viable (Supplementary Fig. 2a,b). All experiments were performed within 5 h of blood collection and isolation.

For stimulation, between 1.5 and 3 million neutrophils were aliquoted into 1.5-ml Eppendorf tubes or plated on Cell-Tak-coated plates. Stimulation by PMA (Cayman Chemical, no. 10008014) was provided to cell suspensions at the indicated concentrations in each experiment. *P. aeruginosa* (PAK strain) were cultured overnight, subcultured until midexponential phase then opsonized in human serum (Sigma-Aldrich, no. H4522) for 30 min. Opsonized PsA was spun down at 10,000*g* for 1 min, washed and resuspended in PBS then added to neutrophil suspension at 10:1 bacterial/neutrophil cells. Zymosan A was opsonized by incubation with human serum (Sigma-Aldrich, no. H4522) at 37 °C for 30 min, washed twice and resuspended in PBS then added to neutrophils plated on Cell-Tak-coated plates at the indicated concentration for stimulation. Either TNF-α (R&D systems, no. 210-TA-020) or fMLP (Cayman Chemical, no. 59880-97-6) was dissolved in PBS to make a stock solution and added to neutrophils on Cell-Tak-coated plates for stimulation. In experiments involving inhibitors, either DPI (Sigma-Aldrich, no. D2926), G6PDi^39^ (obtained from the Rabinowitz laboratory at Princeton University) or 6-AN (Cayman Chemical, no. 329-89-5) was added concurrently with stimulation at the indicated concentrations.

### Assay for oxidative burst

Oxidative burst was measured by stimulation-induced oxygen consumption rate (OCR) using a XF-96e extracellular flux analyser (Agilent) following a protocol developed by Seahorse^71^. To attach neutrophils at the bottom of assay plates (Seahorse Bioscience), neutrophils were plated in culture wells precoated with Cell-Tak (Corning, no. 354240) at 4 × 10^4^ cells per well, spun at 200*g* for 1 min with minimal acceleration/deceleration then incubated for 1 h at 37 °C. Assays were performed in regular neutrophil culture media (RPMI 1640 medium supplemented with 0.1% human serum albumin). Inhibitors DPI, G6PDi, 6-AN or vehicle control were added just before starting the assay. After several baseline OCR measurements, biochemical stimulant was injected through the injection ports to induce oxidative burst, and the OCR was continuously monitored throughout the course of oxidative burst. To measure oxidative burst induced by PsA, opsonized bacteria were added directly to the plate because they could not be consistently injected via the ports, and OCR was measured after 30 min of coincubation. To adjust for oxygen consumption by bacteria, OCR was also measured in parallel wells containing only PsA (same number) but no neutrophils, and this value was then subtracted from coincubation measurements.

### NET release assay

NET release was quantified by increase in extracellular DNA over time following a previously developed protocol^72^. Briefly, neutrophils were plated in at 4 × 10^4^ cells per well in a 96-well tissue culture plate precoated with Cell-Tak (Corning, no. 354240) and spun at 200*g* for 1 min with minimal acceleration/deceleration. Cytotox Green Reagent (IncuCyte, no. 4633) was added to culture media at 1:4,000 to stain extracellular DNA, and images were captured every 10–30 min after stimulation using an IncuCyte live cell imager under standard culture conditions (37 °C, 5% CO_2_). Representative images are shown in Supplementary Fig. 1. The fluorescent area outside of cells, which indicates NET, was quantified by image analysis using IncuCyte S3 Basic Analysis software.

### Metabolomics and isotopic tracing

To extract intracellular metabolites, culture medium was removed and neutrophils were immediately washed with PBS. Each 2 million pelleted neutrophils were extracted with 150 µl of cold liquid chromatography–mass spectrometry (LC–MS)-grade acetonitrile/methanol/water (40:40:20 v:v:v), and samples were spun at 20,627*g* for 5 min at 4 C to remove any insoluble debris. Soluble metabolite samples were analysed with a Thermo Q-Exactive mass spectrometer coupled to a Vanquish Horizon Ultra-High Performance Liquid Chromatograph, using the following two analytical methods. (1) Samples in extraction solvent were directly loaded on to LC–MS, then separated on a 2.1 × 150mm Xbridge BEH Amide (2.5 μm) Column (Waters) using a gradient of solvent A (95% H_2_O, 5% ACN, 20 mM NH_4_AC, 20 mM NH_4_OH) and solvent B (20% H_2_O, 80% ACN, 20 mM NH_4_AC, 20 mM NH_4_OH). The gradient used was 0 min, 100% B; 3 min, 100% B; 3.2 min, 90% B; 6.2 min, 90% B; 6.5 min, 80% B; 10.5 min, 80% B; 10.7 min, 70% B; 13.5 min, 70% B; 13.7 min, 45% B; 16 min, 45% B; 16.5 min, 100% B; 22 min, 100% B. The flow rate was 0.3 ml min^–1^ and column temperature 30 °C. Analytes were measure by MS using full scan. (2) Samples were dried under N_2_ flow and resuspended in LC–MS-grade water as loading solvent. Metabolites were separated on a 2.1 × 100mm, 1.7 µM Acquity UPLC BEH C18 Column (Waters) with a gradient of solvent A (97:3 H_2_O/methanol, 10 mM TBA, 9 mM acetate, pH 8.2) and solvent B (100% methanol). The gradient was: 0 min, 5% B; 2.5 min, 5% B; 17 min, 95% B; 21 min, 95% B; 21.5 min, 5% B. Flow rate was 0.2 ml min^–1^. Data were collected with full scan. Identification of metabolites reported here was based on exact *m/z* and retention time that were determined with chemical standards. Data were collected with Xcalibur 4.0 software and analysed with Maven.

To determine the molar ratio of [F6P]/[G6P], the concentrations of F6P and G6P were quantified using an external calibration curve generated with G6P and F6P standards.

For all experiments involving stable isotope tracers, including 1-^13^C-glucose, 1,2-^13^C-glucose, U-^13^C-glucose and 3-^2^H-glucose (all from Cambridge Isotope), isotopically labelled glucose was substituted for unlabelled glucose at the same concentration in RPMI culture medium. Natural ^13^C abundance was corrected from raw data.

### Metabolic flux analysis

Metabolic flux analysis was performed using the INCA software suite^46^, which is implemented in MATLAB to simulate isotopic distribution using an elementary metabolite unit framework^73^. This estimates intracellular fluxes by solving a non-linear least-squares regression problem that minimizes the variance-weighted sum of squared residuals between simulated and measured isotopic distributions of intracellular metabolites, under the assumption of pseudo-steady state (verified in Extended Data Fig. 3). The network model includes all reactions in glycolysis and PPP, as specified in Supplementary Table 1. Reactions in glycolysis and non-OxPPP are allowed to be reversible. Reversible reactions were modelled as a forward-and-backward reaction. Net fluxes equal forward flux minus backward flux, and exchange flux equals the smaller flux between forward and backward fluxes. Carbon sinks include both lactate and CO_2_. The inclusion of carbon sinks in the model is based on U-^13^C-glucose labelling results, with the rationale that a considerable carbon sink for glucose metabolism would label from U-^13^C-glucose. Other possible carbon sinks, such as serine and glycine, are not included in the model because they label no more than 3% from U-^13^C-glucose within the experimental time frame. Potential low carbon sinking rates from these compounds may have a limited impact on flux analysis results. Glucose influx into the system is fixed at 100, because this analysis is intended to reveal relative metabolic flux distribution relative to total glucose uptake rate. The model also allows input from glycogen (via G1P) based on the biochemical knowledge that neutrophils can use glycogen. All fluxes, except glucose uptake, are completely unconstrained. Labelling data from 1,2-^13^C-glucose tracer experiments, corrected for naturally occurring heavy isotopes, were entered into the model. Data on neutrophils from each donor used in the analysis are listed in Supplementary Table 1. Hexose-phosphate labelling results were not used for fitting in the flux analysis because various hexose-phosphates are not fully resolved on chromatography, and thus measured labelling may be affected by some interference from other hexose-phosphate compounds. The error of input data was set to 2% to reflect the typical technical variation of such LC–MS measurements. Variation from biological sources (that is, donor to donor) can be seen from comparing the fitting results from each donor (Fig. 3d and Extended Data Fig. 3d). Random initial guess for flux estimation was applied and was reinitialized. Using the optimal fitting solution, we calculated 95% confidence intervals for all estimated fluxes by performing parameter continuation analysis. Fitting parameters used in INCA are listed in Supplementary Table 1. Fitting results, including flux results for each donor with upper and lower bound and goodness of fit, are compiled in Supplementary Table 2.

### Culture and genetic KO of HL-60 cells

To generate genetic KO we used HL-60 (ATCC CCL-240) cell lines. HL-60 cells were cultured in RPMI with 5 mM glutamine, 25 mM HEPES, 15% fetal bovine serum (FBS) and 1% penicillin/streptomycin. To differentiate HL-60 to a neutrophil-like state, cells were transferred to differentiation medium (RPMI with 1.3% DMSO, 5 mM glutamine, 25 mM HEPES, 9% FBS and 1% penicillin/streptomycin) for 6 days, with medium change on day 3. After 6 days, differentiated neutrophil-like cells were verified by flow cytometry (Supplementary Fig. 2c,d) using antibody against surface marker CD11B (BioLegend, no. 3013045), and were used in experiments in culture media without DMSO. HL-60 cell lines with *TKT* and *TALDO1* KO were created using the Alt-R RNP system consisting of Alt-R CRISPR–Cas9 transactivating CRISPR RNA, ATTO 550 (5 nmol; IDT, no. 1075927) and Alt-R S.p. HiFi Cas9 Nuclease V3 (100 µg; IDT, no. 1081060), following the manufacturer’s instructions. The following CRISPR RNA sequence was used: TKT, (GACCGGGTGCCCGTCCAAGT), TALDO1 (ACCACCGTGGTGGCCGACAC). After transfection of the RNP complex into cells by electroporation using the Amaxa Nucleofector system Kit V (Lonza, no. VCA-1003), fluorescent cells were sorted into signal cells and expanded in IMDM with 20% FBS. To validate KO in viable single colonies, genomic DNA was extracted using the QIAamp DNA Mini Kit (Qiagen, no. 51304) and a region around the CRISPR cut site target was amplified using the following primers containing M13 tags for sequencing. *TKT*: forward (TGTAAAACGACGGCCAGTTTCTCAGTGGGCACCCCCTAC), reverse (CAGGAAACAGCTATGACCCACAAACCAGGGATTAGGGCAGC); *TALDO1*: forward (TGTAAAACGACGGCCAGTTCGTGCAGGTGTTTTCCCG), reverse (CAGGAAACAGCTATGACCGCTCATTCCTCCCGGAACGA). *TKT* KO was verified by Sanger sequencing (Genewiz) of the PCR product after column purification (Thermo Scientific, no. K0701). The sequencing results, shown in Supplementary Table 3, indicated specific mutations. *TALDO1* KO was indicated by both the specific absence of TADLO1 DNA amplification and quantitative real-time PCR (qPCR) showing lack of expression of TADLO1 (Supplementary Fig. 3). For real-time PCR, RNA was extracted using RNAStat60 (Tel-Test) and treated with RQ1 DNAse (Promega, no. M6101). Complementary DNA was synthesized from DNAse-treated RNA samples (1–2 μg) using SuperScript III (ThermoFisher, no. 12574026), following the manufacturer’s instructions. Quantitative PCR was performed using KAPA SYBR FAST qPCR MasterMix on a Roche LightCycler 480. Relative gene expression level was normalized to the housekeeping gene *β2-Microglobulin*. The following primers (IDT) were used: *TALDO1* Fwd 5'-CTCACCCGTGAAGCGTCAG-3'; *TALDO1* Rev 5'-GTTGGTGGTAGCATCCTGGG-3'. KO cells are cultured and differentiated as for WT.

### Immunoblotting

Approximately 4 million cells were harvested with RIPA lysis buffer with phosphatase inhibitor (Thermo Scientific, no. A32957) and HALT protease inhibitor (Thermo Scientific, no. 78425). Proteins of interest were probed with the following antibodies in TBS-T buffer with 5% BSA. Primary antibodies: G6PD (abcam, no. ab993), TIGAR (abcam, no. ab37910), Beta-actin (Cell Signaling, no. 3700 S); and secondary antibodies: Goat-anti-rabbit 800 (LI-COR, no. 925-32211), Goat-anti-mouse 680 (LI-COR, no. 925-68070).

### Enzyme activity assay

To determine enzyme activity in cell lysates, approximately 10 million HL-60 cells were lysed using hypotonic protein assay buffer (25 mM Tris-HCl pH 7.4, 150 mM NaCl, 1% Triton X-100, 1 mM EDTA, 5% glycerol, phosphatase inhibitor, 3× HALT protease inhibitor). The cell suspension was passed through a 25-G needle three times, then centrifuged at 12,000*g* at 4 °C for 5 min to clear cellular debris. Total protein concentration was determined by BCA assay kit (ThermoFisher, no. J63283-QA).

To measure the maximal NADPH production rate using G6P or F6P as the substrate, 20 µl of cell lysate was diluted to a volume of 50 µl with protein assay buffer (listed above). Substrates (NADP^+^ and G6P, or NADP^+^ and F6P) were added to reach a final concentration of 1 mM for each substrate, and NADPH production was monitored continuously by absorbance at 340 nm using a plate reader (Epoch) over 1 h. The reaction mix, containing cell lysate and 1 mM NADP^+^ but no substrate G6P or F6P, was monitored in parallel as a blank control. At the end of experiment, the reaction mix was quenched by methanol and the products were confirmed by LC–MS analysis. Reaction rates were determined by linear regression of NADPH production (after subtraction of the blank). Slopes were normalized to total protein concentration.

To assess the activity of PFK and aldolase in cell lysates, we measured the production of fructose-1,6-bisphosphate and dihydroxyacetone phosphate from F6P. The reaction was started by mixing cell lysate and substrates (final concentration of 1 mM F6P and 1 mM ATP) in assay buffer. Reactions were quenched at 1, 2, 3 and 4 h using a 4× volume of methanol. The products were quantified by LC–MS using chemical standards as external calibration. Reaction mix with cell lysate and 1 mM ATP, but no F6P, was monitored as blank control. Reaction rates were determined by linear regression of product formation (after subtraction of the blank), and were normalized to cell lysate protein concentrations.

### Measurement of oxidative stress in PsA coincubated with neutrophils

To assess oxidative stress in bacteria phagocytized by neutrophils, we measured reduced to oxidized glutathione ratio in PsA coincubated with neutrophils following a previously described method^55^. Briefly, PsA culture was grown overnight in minimal medium with U-^13^C-glucose as the sole carbon source, then subcultured in labelled medium to midexponential growth phase to fully label bacterial metabolites. Prelabeled PsA was first opsonized, then coincubated with freshly isolated human peripheral blood neutrophils in neutrophil medium (RPMI with HSA) containing U-^13^C-glucose for 30 min. Neutrophils were then pelleted by centrifugation at 500*g* for 3 min and extracellular bacteria were removed. The neutrophil pellet was then washed with PBS and metabolites from neutrophils and intracellular bacteria were extracted. Heavy labelled GSH and GSSG were derived from phagocytized bacteria, whereas GSH and GSSG from neutrophils remained unlabelled because neutrophils did not synthesize considerable amounts of new glutathione from U-^13^C-glucose within 30 min (verified by neutrophil-only control cultured in U-^13^C-glucose RPMI medium for 30 min). The bacterial (labelled) GSH/GSSG ratio was determined by LC–MS.

### Pathogen-killing assay

Pathogen-killing ability was assessed by mixing serum opsonized PsA with human peripheral blood neutrophils or differentiated HL-60 cells at a 10:1 ratio and incubation at 37 °C for 15 min. Neutrophils were pelleted by spinning at 500*g* for 3 min. The pellet was washed with PBS then lysed with 1% saponin + 100 µg ml^–1^ DNase at 37 °C for 10 min. The bacteria were pelleted with spinning for 1 min at 8,000*g*, resuspended in PBS and plated on lysogeny broth agar plates to determine CFU.

### Survival analysis of zebrafish larvae challenged with *A. nidulans*

The experiments with zebrafish (*Danio rerio*, strain AB) were approved by the University of Wisconsin-Madison College of Agricultural and Life Sciences Animal Care and Use Committee (protocol no. M005405-R02). This protocol adheres to the federal Health Research Extension Act and the Public Health Service Policy on the Humane Care and Use of Laboratory Animals, overseen by the National Institutes of Health Office of Laboratory Animal Welfare. The *p22*^*–/–*^zebrafish used in this study contain a mutant *p22*^*phox*^ allele (sa11798) and were obtained from the Zebrafish International Resource Center. WT larvae (*p22*^*+/+*^) used were siblings to *p22*^*–/–*^ larvae.

All *A. nidulans* spore collection and microinjection procedures were performed according to previously established protocols^56^. Specifically, *A. nidulans* (background FGSC-A4, strain TBK100, genotype *pyrG::gpdA::rfp; pyrG89; veA* + *;Δnku::argB*) was grown at 37 °C on solid glucose minimal medium (GMM) for 3 days. *A. nidulans* spores were then collected by scraping with an L-spreader in 0.01% Tween-water, followed by filtration through Miracloth to remove hyphal debris. The spore suspension was centrifuged at 900*g* for 10 min at room temperature. The spore pellet was then resuspended in 50 ml of 1× PBS and passed through a vacuum filter with a Buchner filter funnel and a glass disk with 10–15-μm-diameter pores. The spore suspension was then centrifuged again at 900*g* for 10 min at room temperature and the pellet resuspended in 1 ml of 1× PBS. Spores were counted using a hemacytometer and the concentration adjusted to 1.5 × 10^8^ spores ml^–1^, and were then stored at 4°C for up to 1 month.

For microinjection, 2-day-post-fertilization larvae (*p22*^*–/–*^ or *p22*^*+/+*^) were anesthetized with 0.2 mg ml^–1^ Tricaine (Sigma), and 3 nl of *A. nidulans* spore inoculum was injected into the hindbrain ventricle via the otic vesicle using a capillary needle attached to a Picospritzer microinjector. The spore inoculum was mixed in a 2:1 ratio with 1% phenol red to visualize inoculum in the hindbrain. Following injection (~20 larvae per condition), larvae were rinsed with E3 without methylene blue (E3-MB) to remove Tricaine and larvae were transferred to 35-mm Petri dishes. Larvae were treated by the addition to the dish of 4 ml of either 1 mM 6-AN or 1% DMSO control in E3-MB. Larvae were then transferred to individual wells of a 96-well plate and survival was monitored daily up to 4 days after infection. Larval medium with fresh 6-AN or DMSO solution was exchanged each day. To determine the average number of spores injected, eight larvae from each condition (*p22*^*–/–*^ or *p22*^*+/+*^) were collected immediately following injection, homogenized and plated on solid GMM. Plates were incubated for 3 days at 37 °C and CFU counted. Average spore doses were 47 (*p22*^*–/–*^) and 52 (*p22*^*+/+*^).

### MSU peritonitis model

This study was approved by the Boston Children’s Hospital Institutional Animal Care and Use Committee (protocol no. 00001290). Eight-week-old C57/BL6 female mice (Jackson Laboratories) were given 0.5 mg of synthesized MSU crystal^57^ in 200 µl of PBS intraperitoneally and analysed after 24 h. Neutrophils from peritoneal lavage and bone marrow were isolated by negative selection using the EasySep Neutrophil Isolation Kit (Stem Cell Technologies), according to the manufacturer’s instructions. Isolated neutrophils (2 × 10^6^ per sample) were quenched with ice-cold 80% methanol in preparation for metabolomic studies.

### Metabolic analysis in macrophages

Bone marrow-derived macrophages (BMDMs) isolated from WT C57Bl/6 J mice or *NOS2*^–/–^ mice (Jackson Laboratory) were used for macrophage experiments. Mice were bred and maintained according to protocols approved by the University of Wisconsin-Madison Institutional Animal Care and Use Committee. Bone marrow cells were harvested from femora and tibia of male and female 6–10-week-old mice. Cells were differentiated in RPMI 1640 containing 25 mM HEPES, 1% penicillin/ streptomycin, 10% FBS and 20% L929 conditioned media. Three days after isolation, media were changed every day subsequently up to day 7 after isolation. Cells were then plated for experiments at 5 × 10^5^ per well in six-well plates in RPMI 1640 containing 25 mM HEPES, 1% penicillin/streptomycin, 10% dialyzed FBS and 20 ng ml^–1^ macrophage colony-stimulating factor (R&D Systems). To stimulate BMDMs, cells were incubated with 50 ng ml^–1^ LPS (*E. coli* O111:B4, Sigma) and 30 ng ml^–1^ IFN-γ (R&D Systems). To avoid nutrient depletion and maintain LPS and IFN-γ, media were refreshed daily with LPS and IFN-γ in the media throughout.

### Statistics and reproducibility

Statistical analyses were conducted using GraphPad software, with each statistical test listed in the figure legends. The majority of experiments were conducted at least twice to ensure reproducibility, as detailed in figure legends and Extended Data figure legends. Experimental sample sizes used are noted in the figure legends. No statistical method was used to predetermine sample size. In some cases, sample sizes in the same figure across conditions were different mainly due to variation in neutrophil yield from isolation with each blood draw, which can be limited to cover all experimental conditions. Also, due to limited quantity of samples, in a few cases the measurement of certain compounds had only one technical replicate instead of two (as indicated in Source data) because there were insufficient samples to inject twice in both LC–MS methods covering the measurement of all compounds. In most experiments no technically successful measurements were excluded from analyses, with two exceptions: in Fig. 1f one outlier was removed based on the Grubbs test, and in Fig. 6a one outlier in the graph for 6PG was removed based on the Grubbs test (*α* = 0.05). Samples are randomly assigned to experimental conditions. The investigators were not blinded to the experiment.

### Reporting Summary

Further information on research design is available in the Nature Research Reporting Summary linked to this article.

## Supplementary information

Supplementary Information Supplementary Table 3 and Figs. 1–3.

Reporting Summary

Supplementary Table 1 Metabolic flux analysis model, INCA fitting parameters and input.

Supplementary Table 2 Flux analysis results.

## Data Availability

The data that support the findings of this study are available in source data files linked to each figure.

## Source data

Source Data Fig. 1 Statistical source data.
Source Data Fig. 2 Statistical source data.
Source Data Fig. 3 Statistical source data.
Source Data Fig. 4 Statistical source data.
Source Data Fig. 5 Statistical source data
Source Data Fig. 6 Statistical source data.
Source Data Extended Data Fig. 1 Statistical source data.
Source Data Extended Data Fig. 2 Statistical source data.
Source Data Extended Data Fig. 3 Statistical source data
Source Data Extended Data Fig. 4 Statistical source data.
Source Data Extended Data Fig. 5 Statistical source data.
Source Data Extended Data Fig. 5 Unprocessed immunoblots.
Source Data Extended Data Fig. 6 Statistical source data.

## Code availability

No custom code was used in this paper. LC–MS data analysis and metabolic flux analysis were performed using published software, as indicated in Methods.

## References

[1] de Oliveira S, Rosowski EE, Huttenlocher A (2016). Neutrophil migration in infection and wound repair: going forward in reverse. Nat. Rev. Immunol..

[2] Brinkmann V (2004). Neutrophil extracellular traps kill bacteria. Science.

[3] Mayadas TN, Cullere X, Lowell CA (2014). The multifaceted functions of neutrophils. Annu. Rev. Pathol. Mech. Dis..

[4] Nguyen GT, Green ER, Mecsas J (2017). Neutrophils to the ROScue: mechanisms of NADPH oxidase activation and bacterial resistance. Front. Cell. Infect. Microbiol..

[5] Fuchs TA (2007). Novel cell death program leads to neutrophil extracellular traps. J. Cell Biol..

[6] Reeves EP (2002). Killing activity of neutrophils is mediated through activation of proteases by K^+^ flux. Nature.

[7] Winterbourn CC, Kettle AJ, Hampton MB (2016). Reactive oxygen species and neutrophil function. Annu. Rev. Biochem..

[8] Dale DC, Boxer L, Conrad Liles W (2008). The phagocytes: neutrophils and monocytes. Blood.

[9] Burn GL, Foti A, Marsman G, Patel DF, Zychlinsky A (2021). The neutrophil. Immunity.

[10] O’Neill LAJ, Kishton RJ, Rathmell J (2016). A guide to immunometabolism for immunologists. Nat. Rev. Immunol..

[11] Klein Geltink RI, Kyle RL, Pearce EL (2018). Unraveling the complex interplay between T cell metabolism and function. Annu. Rev. Immunol..

[12] Poznanski SM, Barra NG, Ashkar AA, Schertzer JD (2018). Immunometabolism of T cells and NK cells: metabolic control of effector and regulatory function. Inflamm. Res..

[13] Olenchock BA, Rathmell JC, Vander Heiden MG (2017). Biochemical underpinnings of immune cell metabolic phenotypes. Immunity.

[14] Pearce EL, Pearce EJ (2013). Metabolic pathways in immune cell activation and quiescence. Immunity.

[15] Ryan DG, O’Neill LAJ (2020). Krebs cycle reborn in macrophage immunometabolism. Annu. Rev. Immunol..

[16] Maciver NJ, Michalek RD, Rathmell JC (2013). Metabolic regulation of T lymphocytes. Annu. Rev. Immunol..

[17] O’Brien KL, Finlay DK (2019). Immunometabolism and natural killer cell responses. Nat. Rev. Immunol..

[18] Kumar S, Dikshit M (2019). Metabolic insight of neutrophils in health and disease. Front. Immunol..

[19] Rice CM (2018). Tumour-elicited neutrophils engage mitochondrial metabolism to circumvent nutrient limitations and maintain immune suppression. Nat. Commun..

[20] Sadiku, P. et al. Neutrophils fuel effective immune responses through gluconeogenesis and glycogenesis. *Cell Metab*. **33**, 411–423 (2021). 10.1016/j.cmet.2020.11.016 PMC7863914 33306983

[21] Kuehne, A. et al. Acute activation of oxidative pentose phosphate pathway as first-line response to oxidative stress in human skin cells. *Mol. Cell*10.1016/j.molcel.2015.06.017 (2015). 10.1016/j.molcel.2015.06.017 26190262

[22] Christodoulou, D. et al. Reserve flux capacity in the pentose phosphate pathway enables *Escherichia coli*’s rapid response to oxidative stress. *Cell Syst*. 10.1016/j.cels.2018.04.009 (2018). 10.1016/j.cels.2018.04.009 29753645

[23] Stincone A (2015). The return of metabolism: biochemistry and physiology of the pentose phosphate pathway. Biol. Rev..

[24] Lewis CA (2014). Tracing compartmentalized NADPH metabolism in the cytosol and mitochondria of mammalian cells. Mol. Cell.

[25] DeBerardinis RJ (2007). Beyond aerobic glycolysis: transformed cells can engage in glutamine metabolism that exceeds the requirement for protein and nucleotide synthesis. Proc. Natl Acad. Sci. USA.

[26] Liu L (2016). Malic enzyme tracers reveal hypoxia-induced switch in adipocyte NADPH pathway usage. Nat. Chem. Biol..

[27] Jiang, L. et al. Reductive carboxylation supports redox homeostasis during anchorage-independent growth. *Nature* **532**, 255–258 (2016). 10.1038/nature17393 PMC4860952 27049945

[28] Zhang GF (2021). Reductive TCA cycle metabolism fuels glutamine- and glucose-stimulated insulin secretion. Cell Metab..

[29] Zhang, Z. et al. Serine catabolism generates liver NADPH and supports hepatic lipogenesis. *Nat. Metab.* **3**, 1608–1620 (2021). 10.1038/s42255-021-00487-4 PMC8721747 34845393

[30] Piskounova E (2015). Oxidative stress inhibits distant metastasis by human melanoma cells. Nature.

[31] Christodoulou D (2019). Reserve flux capacity in the pentose phosphate pathway by NADPH binding Is conserved across kingdoms. iScience.

[32] Ben‐Yoseph O, Camp DM, Robinson TE, Ross BD (1995). Dynamic measurements of cerebral pentose phosphate pathway activity in vivo using [1,6‐13C2,6,6‐2H2] glucose and microdialysis. J. Neurochem..

[33] Ralser, M. et al. Dynamic rerouting of the carbohydrate flux is key to counteracting oxidative stress. *J. Biol*. **6**, 10 (2007). 10.1186/jbiol61 PMC2373902 18154684

[34] Yoshida A, Lin M (1973). Regulation of glucose 6 phosphate dehydrogenase activity in red blood cells from hemolytic and nonhemolytic variant subjects. Blood.

[35] Daneshmandi S (2021). Blockade of 6-phosphogluconate dehydrogenase generates CD8^+^ effector T cells with enhanced anti-tumor function. Cell Rep..

[36] Baardman J (2018). A defective pentose phosphate pathway reduces inflammatory macrophage responses during hypercholesterolemia. Cell Rep..

[37] Klein Geltink, R. I. et al. Metabolic conditioning of CD8^+^ effector T cells for adoptive cell therapy. *Nat. Metab.* **2**, 703–716 (2020). 10.1038/s42255-020-0256-z PMC10863625 32747793

[38] Azevedo EP (2015). A metabolic shift toward pentose phosphate pathway is necessary for amyloid fibril- and phorbol 12-myristate 13-acetate-induced neutrophil extracellular trap (NET) formation. J. Biol. Chem..

[39] Ghergurovich JM (2020). A small molecule G6PD inhibitor reveals immune dependence on pentose phosphate pathway. Nat. Chem. Biol..

[40] Amara N (2021). Selective activation of PFKL suppresses the phagocytic oxidative burst. Cell.

[41] Mantovani A, Cassatella MA, Costantini C, Jaillon S (2011). Neutrophils in the activation and regulation of innate and adaptive immunity. Nat. Rev. Immunol..

[42] Malhotra, S., Hayes, D. & Wozniak, D. J. Cystic fibrosis and pseudomonas aeruginosa: the host-microbe interface. *Clin. Microbiol. Rev*. **32**, e00138-18 (2019). 10.1128/CMR.00138-18 PMC6589863 31142499

[43] Katz, J. & Wood, H. G. The use of glucose-C14 for the evaluation of the pathways of glucose metabolism. *J. Biolog. Chem.* **235**, 2165–2177 (1960). 14404802

[44] Dick TP, Ralser M (2015). Metabolic remodeling in times of stress: who shoots faster than his shadow?. Mol. Cell.

[45] Lee, W. N. P. et al. Mass isotopomer study of the nonoxidative pathways of the pentose cycle with [1,2-13C2]glucose. *Am. J. Physiol.* **274**, E843–E851 (1998). 10.1152/ajpendo.1998.274.5.E843 9612242

[46] Young JD (2014). INCA: a computational platform for isotopically non-stationary metabolic flux analysis. Bioinformatics.

[47] Yoshida A, Lin M (1973). Regulation of glucose-6-phosphate dehydrogenase activity in red blood cells from hemolytic and nonhemolytic variant subjects. Blood.

[48] Holten D, Procsal D, Chang HL (1976). Regulation of pentose phosphate pathway dehydrogenases by NADP^+^ NADPH ratios. Biochem. Biophys. Res. Commun..

[49] Cracan V, Titov DV, Shen H, Grabarek Z, Mootha VK (2017). A genetically encoded tool for manipulation of NADP^+^/NADPH in living cells. Nat. Chem. Biol..

[50] Panday A, Sahoo MK, Osorio D, Batra S (2015). NADPH oxidases: an overview from structure to innate immunity-associated pathologies. Cell. Mol. Immunol..

[51] Rabani R, Cossette C, Graham F, Powell WS (2020). Protein kinase C activates NAD kinase in human neutrophils. Free Radic. Biol. Med..

[52] Hua, A. B. et al. Repurposing the electron transfer reactant phenazine methosulfate (Pms) for the apoptotic elimination of malignant melanoma cells through induction of lethal oxidative and mitochondriotoxic stress. *Cancers (Basel)* **11**, 590 (2019). 10.3390/cancers11050590 PMC6562717 31035569

[53] Peralta D (2015). A proton relay enhances H_2_O_2_ sensitivity of GAPDH to facilitate metabolic adaptation. Nat. Chem. Biol..

[54] Bensaad, K. et al. TIGAR, a p53-inducible regulator of glycolysis and apoptosis. *Cell*10.1016/j.cell.2006.05.036 (2006). 10.1016/j.cell.2006.05.036 16839880

[55] Flamholz A, Noor E, Bar-Even A, Milo R (2012). EQuilibrator – the biochemical thermodynamics calculator. Nucleic Acids Res..

[56] Dickerhof, N. et al. Exposure of *Pseudomonas aeruginosa* to bactericidal hypochlorous acid during neutrophil phagocytosis is compromised in cystic fibrosis. *J. Biol. Chem*. **294**, 13502–13514 (2019). 10.1074/jbc.RA119.009934 PMC6737234 31341024

[57] Schoen, T. J. et al. Neutrophil phagocyte oxidase activity controls invasive fungal growth and inflammation in zebrafish. *J. Cell Sci*. **133**, jcs236539 (2019). 10.1242/jcs.236539 PMC7055366 31722976

[58] Martin WJ, Walton M, Harper J (2009). Resident macrophages initiating and driving inflammation in a monosodium urate monohydrate crystal–induced murine peritoneal model of acute gout. Arthritis Rheum..

[59] Cinelli MA, Do HT, Miley GP, Silverman RB (2020). Inducible nitric oxide synthase: regulation, structure, and inhibition. Med. Res. Rev..

[60] Berg, J., Tymoczko, J. & Stryer, L. *Biochemistry* 6th edn (W.H. Freeman Company) (2006).

[61] Patra KC, Hay N (2014). The pentose phosphate pathway and cancer. Trends Biochem. Sci..

[62] Ying H (2012). Oncogenic Kras maintains pancreatic tumors through regulation of anabolic glucose metabolism. Cell.

[63] Boros LG (2005). [1,2-13C2]-D-glucose profiles of the serum, liver, pancreas, and DMBA-induced pancreatic tumors of rats. Pancreas.

[64] Brekke E, Morken TS, Sonnewald U (2015). Glucose metabolism and astrocyte-neuron interactions in the neonatal brain. Neurochem. Int..

[65] Jalloh I (2015). Glycolysis and the pentose phosphate pathway after human traumatic brain injury: microdialysis studies using 1,2-13C2 glucose. J. Cereb. Blood Flow. Metab..

[66] Fan, J. et al. Quantitative flux analysis reveals folate-dependent NADPH production. *Nature* **510**, 298–302 (2014). 10.1038/nature13236 PMC4104482 24805240

[67] Williams M (2019). Clinical, biochemical, and molecular overview of transaldolase deficiency and evaluation of the endocrine function: update of 34 patients. J. Inherit. Metab. Dis..

[68] Siler U (2017). Severe glucose-6-phosphate dehydrogenase deficiency leads to susceptibility to infection and absent NETosis. J. Allergy Clin. Immunol..

[69] Rodríguez-Espinosa O, Rojas-Espinosa O, Moreno-Altamirano MMB, López-Villegas EO, Sánchez-García FJ (2015). Metabolic requirements for neutrophil extracellular traps formation. Immunology.

[70] Maratou E (2007). Glucose transporter expression on the plasma membrane of resting and activated white blood cells. Eur. J. Clin. Invest..

[71] Swain, P., Romero, N. & Dranka, B. P. Modulation of oxidative burst with exposure to cytokines in neutrophil cell activation. *J. Immunol.* https://www.jimmunol.org/content/200/1_Supplement/49.26 (2018).

[72] Gupta S, Chan DW, Zaal KJ, Kaplan MJ (2018). A high-throughput real-time imaging technique to quantify NETosis and distinguish mechanisms of cell death in human neutrophils. J. Immunol..

[73] Antoniewicz MR, Kelleher JK, Stephanopoulos G (2007). Elementary metabolite units (EMU): a novel framework for modeling isotopic distributions. Metab. Eng..

