# Supplementary material for: Switching to the cyclic pentose phosphate pathway powers the oxidative burst in activated neutrophils
Source: Nat Metab. 2022 Mar 28;4(3):389–403. doi: 10.1038/s42255-022-00550-8 (PMC8964420; doi:10.1038/s42255-022-00550-8)
Supplement: Supplementary file 1 — Supplementary Table 3 and Figs. 1–3. [file 42255_2022_550_MOESM1_ESM.pdf]

nature portfolio

https://doi.org/10.1038/s42255-022-00550-8

Supplementary information

Switching to the cyclic pentose phosphate pathway powers the oxidative burst in activated neutrophils

In the format provided by the
authors and unedited

Supplementary Table

Table S3. Sequence information of CRISPR-edited HL60 cells.
Wilde-type and TALDO1- or TKT- knock out colonies of HL-60 cells used in the manuscript were sequenced after PCR amplification of the target genes using primers specified in method.

TKT gene amplicon

Wild-type	TKT KO (two mutated alleles)	
	Allele 1: 513_514insT	Allele 2: 515_522delins
CTGCCTGCTGGGGTTTTGGCTTCGTCCAGC<br>CTCACAGCTGTGCCCCCTGCACCTGTCTTA<br>GTGCCTGCCTGGCCTTGGCAGTGGGAACTT<br>TGGGCTGAAAACCAAGAGGCTGAAGAGGG<br>CTGAAAGGGGAGAAGTGGCCTTCACTTGCT<br>TCCTCCCCTGAAATGGATGCTGCAGTTGGC<br>CGGCTCGGCCGGGCCCCAGGGGTCAGTGT<br>GAAGTAGGCACAGCCCGAAGTAGAACCGCT<br>CCCCACCTGACCTCACCCCACCATCCATCT<br>CCCGGCAGGGCCATGCAGCTCCCATCCTCT<br>ACGCGGTCTGGGCTGAAGCTGGTTTCCTGG<br>CCGAGGCGGAGCTGCTGAACCTGAGGAAG<br>ATCAGCTCCGACTTGGACGGGCACCCGGTC<br>CCGGTAAGTGCTCCTGCCAGAGCCTCCCAT<br>GCCTCTGCCTCCACTTCCTA	CTGCCTGCTGGGGTTTTGGCTTCGTCC<br>AGCCTCACAGCTGTGCCCCCTGCACCT<br>GTCTTAGTGCCTGCCTGGCCTTGGCAG<br>TGGGAACTTTGGGCTGAAAACCAAGAG<br>GCTGAAGAGGGCTGAAAGGGGAGAAG<br>TGGCCTTCACTTGCTTCCTCCCCTGAA<br>ATGGATGCTGCAGTTGGCCGGCTCGG<br>CCGGGCCCCAGGGGTCAGTGTGAAGT<br>AGGCACAGCCCGAAGTAGAACCGCTC<br>CCCACCTGACCTCACCCCACCATCCAT<br>CTCCCGGCAGGGCCATGCAGCTCCCA<br>TCCTCTACGCGGTCTGGGCTGAAGCTG<br>GTTTCCTGGCCGAGGCGGAGCTGCTG<br>AACCTGAGGAAGATCAGCTCCGAC**T**TT<br>GGACGGGCACCCGGTCCCGGTAAGTG<br>CTCCTGCCAGAGCCTCCCATGCCTCTG<br>CCTCCACTTCCTA	CTGCCTGCTGGGGTTTTGGCTTCGTCCAGCC<br>TCACAGCTGTGCCCCCTGCACCTGTCTTAGTG<br>CCTGCCTGGCCTTGGCAGTGGGAACTTTGGG<br>CTGAAAACCAAGAGGCTGAAGAGGGCTGAAA<br>GGGGAGAAGTGGCCTTCACTTGCTTCCTCCC<br>CTGAAATGGATGCTGCAGTTGGCCGGCTCGG<br>CCGGGCCCCAGGGGTCAGTGTGAAGTAGGCA<br>CAGCCCGAAGTAGAACCGCTCCCCACCTGAC<br>CTCACCCCACCATCCATCTCCCGGCAGGGCC<br>ATGCAGCTCCCATCCTCTACGCGGTCTGGGC<br>TGAAGCTGGTTTCCTGGCCGAGGCGGAGCTG<br>CTGAACCTGAGGAAGATCAGCTCCGACT**ATC**<br>**CATCTCCCGGCAGGGCCATGCAGCTCCCATC**<br>**CTCTACGCGGTCTGGGCTGAAGCTGGTTTCC**<br>**TGGCCGAGGCGGAGCTGCTGAACCTGAGGT**<br>**TCC**CACCCGGTCCCGGTAAGTGCTCCTGCCA<br>GAGCCTCCCATGCCTCTGCCTCCACTTCCTA

TALDO1 gene amplicon

Wild-type	TALDO KO
CTGCCTGCTGGGGTTTTGGCTTCGTCCAGCCTCACAGCTGTGCCCCCTGCACCTGTCTTAGTGCCTGCCTGGCCTTGGCAGT<br>GGGAACTTTGGGCTGAAAACCAAGAGGCTGAAGAGGGCTGAAAGGGGAGAAGTGGCCTTCACTTGCTTCCTCCCCTGAAATG<br>GATGCTGCAGTTGGCCGGCTCGGCCGGGCCCCAGGGGTCAGTGTGAAGTAGGCACAGCCCGAAGTAGAACCGCTCCCCAC<br>CTGACCTCACCCCACCATCCATCTCCCGGCAGGGCCATGCAGCTCCCATCCTCTACGCGGTCTGGGCTGAAGCTGGTTTCCT<br>GGCCGAGGCGGAGCTGCTGAACCTGAGGAAGATCAGCTCCGACTTGGACGGGCACCCGGTCCCGGTAAGTGCTCCTGCCA<br>GAGCCTCCCATGCCTCTGCCTCCACTTCCTA	No amplification

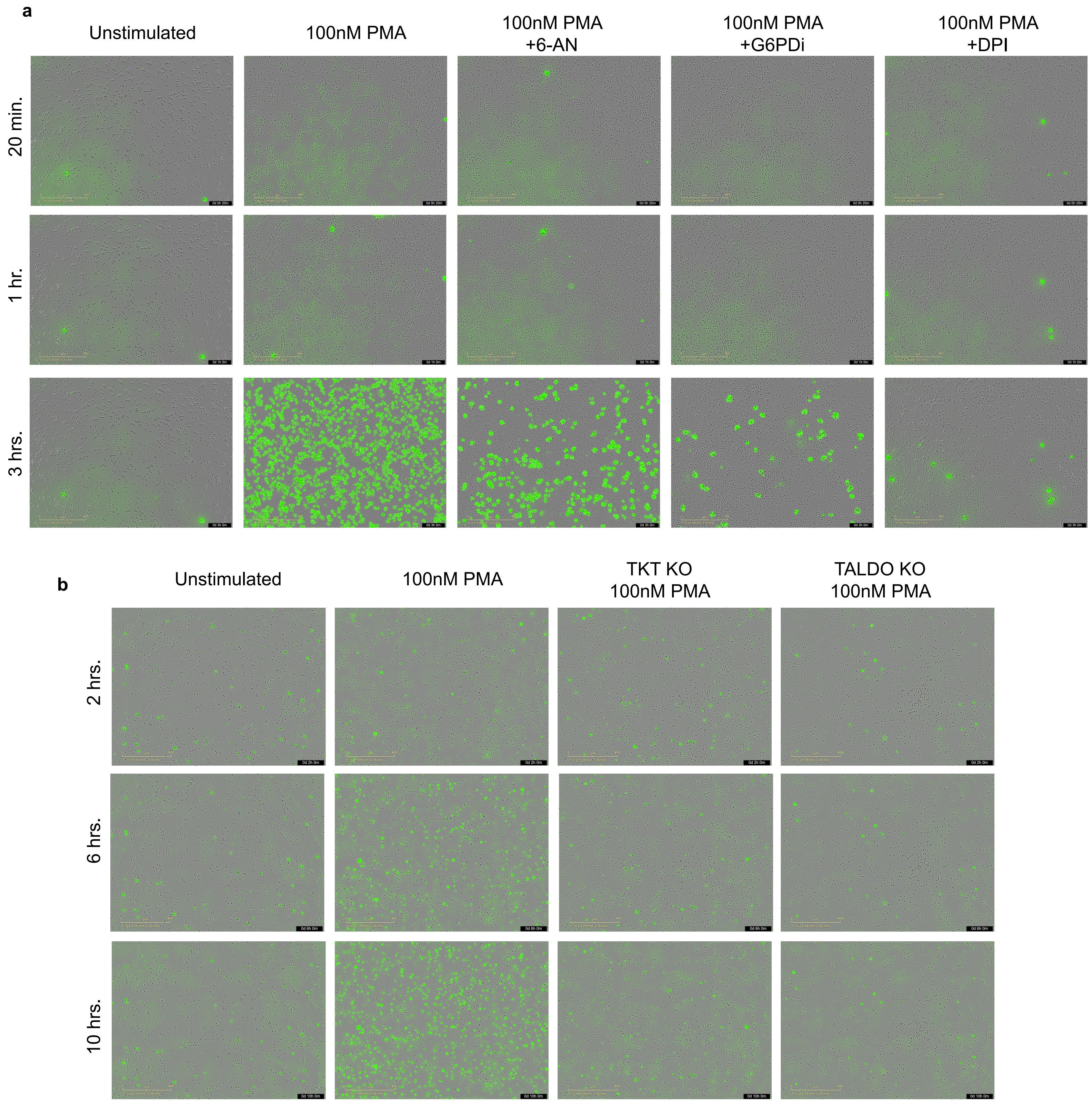

Supplementary Figure 1. NET release relies on PPP activity. Related to Figure 5.
a. Representative images of NET release by human peripheral blood neutrophils stimulated with 100nM PMA, with or without treatment of 6-AN (5mM), G6PDi (50μM), or DPI (10μM), at selected time points.
b. Representative images of NET release by HL-60 cells (wild-type, TKT or TALDO1 knockout) stimulated with 100nM PMA at selected time points. NET is indicated by extracellular DNA, which is stained by green fluorescent dye. NET release was quantified and presented in Fig 5.

a **b**

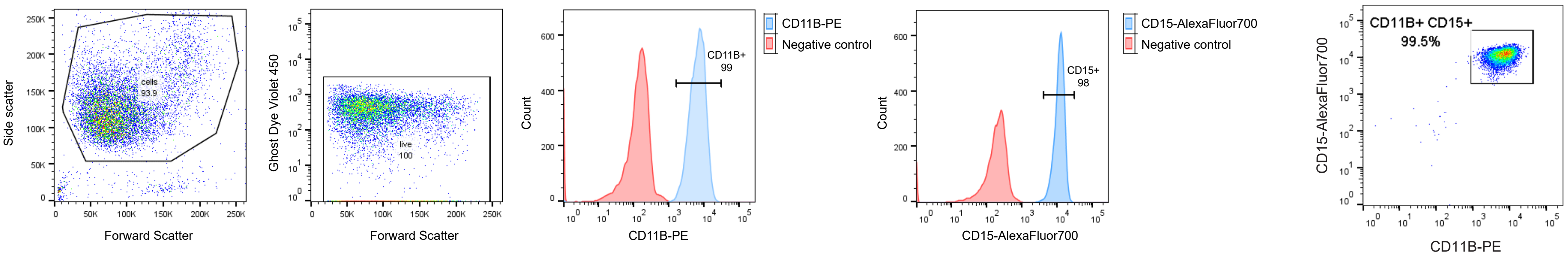

c

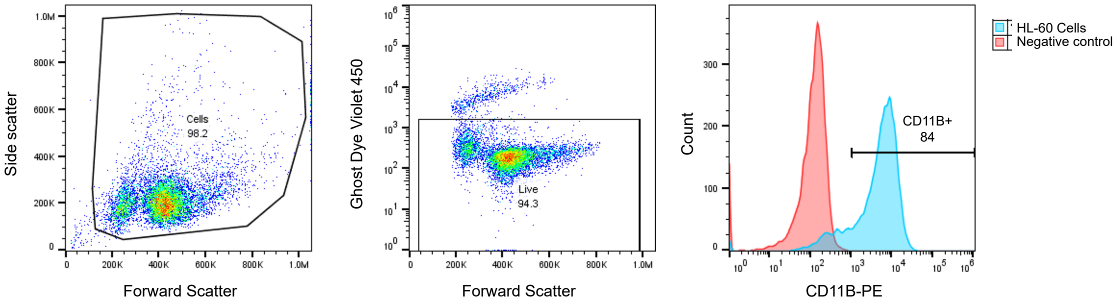

d

Percent CD11B+

	Undifferentiated	Differentiated
WT	20	84
TKT KO	14	85
TALDO1 KO	15	74

Supplementary Figure 2. Quality control of isolated human peripheral blood neutrophils and HL-60 cells.
a. Gating strategy for checking the quality of isolated human peripheral blood neutrophils. Isolated cells were stained with Ghost Dye Violet 450, CD11B-PE, and CD15-AlexaFluor700. b. Purity of isolated human peripheral blood neutrophils c. To check the viability and identity of differentiated HL-60 cells, cells were stained with Ghost Dye Violet 450 and CD11B-PE. Graphs present gating strategy and viability. d. Fraction of cells positive for surface marker CD11B in Wild-type, TKT KO or TALDO1 KO HL-60 cells, before and after differentiation.

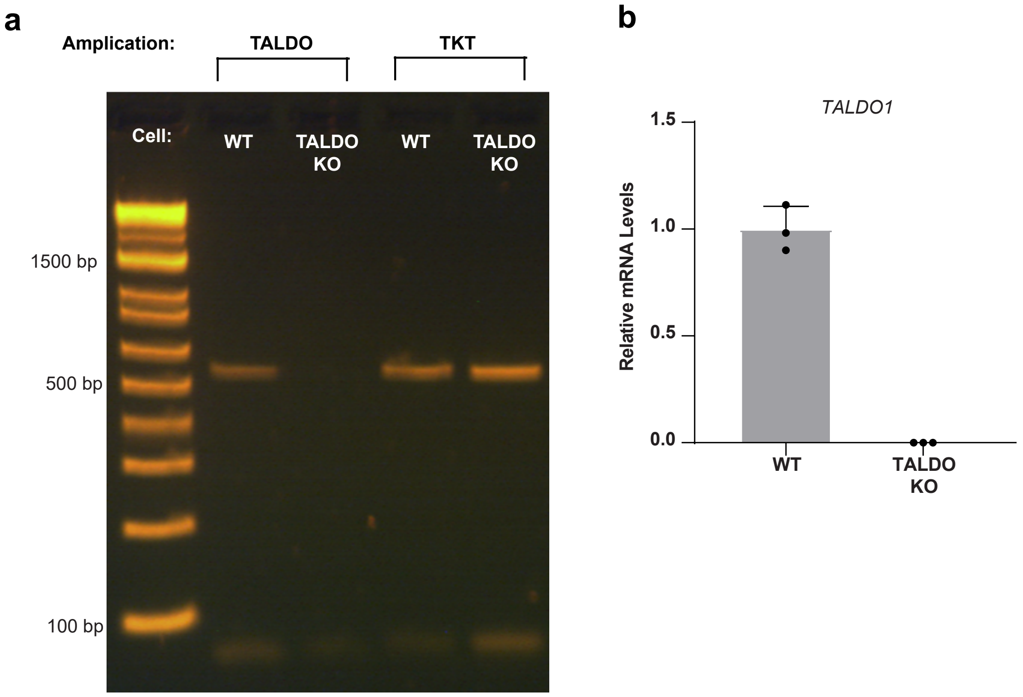

Supplementary Figure 3. Validation of TALDO1 knock out in HL-60 cells
a. DNA was extracted from wild-type or TALDO1 knock out HL-60 cells. A region of TALDO1 or TKT gene was amplified using the primers specified in Method. While TALDO1 was amplified in WT, it failed to amplify in TALDO1 knock out cells. As a control, TKT gene was successfully amplified in both wild type and TALDO1 knock out cells. Results have been confirmed in two independent experiments.
b. Validation of TALDO1 knock out in HL-60 cells determined by qPCR for TALDO1 expression. Mean ± SD from N=3 technical replicates.
