# Supplementary material for: Switching to the cyclic pentose phosphate pathway powers the oxidative burst in activated neutrophils
Source: Nat Metab. 2022 Mar 28;4(3):389–403. doi: 10.1038/s42255-022-00550-8 (PMC8964420; doi:10.1038/s42255-022-00550-8)
Supplement: Supplementary file 2 — Reporting Summary [file 42255_2022_550_MOESM2_ESM.pdf]

nature portfolio

Corresponding author(s): Jing Fan

Last updated by author(s): Feb 5, 2022

Reporting Summary

Nature Portfolio wishes to improve the reproducibility of the work that we publish. This form provides structure for consistency and transparency in reporting. For further information on Nature Portfolio policies, see our Editorial Policies and the Editorial Policy Checklist.

Statistics

For all statistical analyses, confirm that the following items are present in the figure legend, table legend, main text, or Methods section.

n/a	Confirmed	
☐	☒	The exact sample size (*n*) for each experimental group/condition, given as a discrete number and unit of measurement
☐	☒	A statement on whether measurements were taken from distinct samples or whether the same sample was measured repeatedly
☐	☒	The statistical test(s) used AND whether they are one- or two-sided<br>*Only common tests should be described solely by name; describe more complex techniques in the Methods section.*
☒	☐	A description of all covariates tested
☒	☐	A description of any assumptions or corrections, such as tests of normality and adjustment for multiple comparisons
☐	☒	A full description of the statistical parameters including central tendency (e.g. means) or other basic estimates (e.g. regression coefficient) AND variation (e.g. standard deviation) or associated estimates of uncertainty (e.g. confidence intervals)
☐	☒	For null hypothesis testing, the test statistic (e.g. *F*, *t*, *r*) with confidence intervals, effect sizes, degrees of freedom and *P* value noted<br>*Give P values as exact values whenever suitable.*
☒	☐	For Bayesian analysis, information on the choice of priors and Markov chain Monte Carlo settings
☒	☐	For hierarchical and complex designs, identification of the appropriate level for tests and full reporting of outcomes
☒	☐	Estimates of effect sizes (e.g. Cohen's *d*, Pearson's *r*), indicating how they were calculated

Our web collection on statistics for biologists contains articles on many of the points above.

Software and code

Policy information about availability of computer code

Data collection	XCalibur 4.0 software for LCMS data; Agilent XF96 Seahorse 1.4.2.3. for OCR data; IncuCyte S3 2019A for NET release
Data analysis	Graph Pad Prism 7.0 were used to perform statistical analyses; Maven version 6.2 was used to analyze LCMS data (build 682); FlowJo 10.4 (TreeStar) was used for flow cytometry; INCA software suite version 2.0 was used for isotopic labeling analysis; IncuCyte S3 Basic Analysis was used for NET release

For manuscripts utilizing custom algorithms or software that are central to the research but not yet described in published literature, software must be made available to editors and reviewers. We strongly encourage code deposition in a community repository (e.g. GitHub). See the Nature Portfolio guidelines for submitting code & software for further information.

Data

Policy information about availability of data

All manuscripts must include a data availability statement. This statement should provide the following information, where applicable:

- Accession codes, unique identifiers, or web links for publicly available datasets
- A description of any restrictions on data availability
- For clinical datasets or third party data, please ensure that the statement adheres to our policy

Source data are provided with this paper.

Field-specific reporting

Please select the one below that is the best fit for your research. If you are not sure, read the appropriate sections before making your selection.

☒ Life sciences ☐ Behavioural & social sciences ☐ Ecological, evolutionary & environmental sciences

For a reference copy of the document with all sections, see nature.com/documents/nr-reporting-summary-flat.pdf

Life sciences study design

All studies must disclose on these points even when the disclosure is negative.

Sample size	Experimental sample sizes used are noted in the figure legends. No statistical method was used to predetermine sample size.
Data exclusions	In most experiment, no technically successful measurements were excluded from the analyses with the two exceptions: in Fig 1f one outlier is removed based on Grubbs test, in Fig 6a, one outlier in graph for 6PG was removed based on Grubbs test (alpha=0.05).
Replication	Majority of the experiments are successfully conducted at least twice to ensure reproducibility. Specific replication information for each experiment is detailed in figure and extended data figure legends.
Randomization	Cells and animals were randomly assigned to experimental conditions.
Blinding	The investigators were not blinded to the experiment.

Reporting for specific materials, systems and methods

We require information from authors about some types of materials, experimental systems and methods used in many studies. Here, indicate whether each material, system or method listed is relevant to your study. If you are not sure if a list item applies to your research, read the appropriate section before selecting a response.

Materials & experimental systems

n/a	Involved in the study	
☐	☒	Antibodies
☐	☒	Eukaryotic cell lines
☒	☐	Palaeontology and archaeology
☐	☒	Animals and other organisms
☐	☒	Human research participants
☒	☐	Clinical data
☒	☐	Dual use research of concern

Methods

n/a	Involved in the study	
☒	☐	ChIP-seq
☐	☒	Flow cytometry
☒	☐	MRI-based neuroimaging

Antibodies

Antibodies used	Primary Antibodies:<br>CD11b (Biolegend cat. 301305) 1:60 dilution<br>CD15 (Biolegend cat. 301919) 1:60 dilution<br>G6PD (abcam ab993) 1:1000 dilution<br>TIGAR (abcam ab37910) 1:1000 dilution<br>Beta-actin (Cell Signaling 3700S) 1:1000 dilution<br>Secondary antibodies:<br>Goat-anti-rabbit 800 (LI-COR 925-32211) 1:10,000 dilution<br>Goat-anti-mouse 680 (LI-COR 925-68070) 1:10,000 dilution
Validation	All antibodies are commercially available and were commercially validated.<br>CD11b (Biolegend cat. 301305) was validated by the manufacturer in human peripheral blood granulocytes.<br>CD15 (Biolegend cat. 301919) was validated by the manufacturer in human peripheral blood granulocytes.<br>G6PD (abcam ab993) was validated from whole cell lysate of NIH/3T3 cells.<br>TIGAR (abcam ab37910) was validated from whole cell lysate of HeLa, Jurkat, A-431, HEK-293, Hep G2, and MCF-7 cells.<br>Beta-actin (Cell Signaling 3700S) was validated from whole cell lysate of HeLa, C2C12, and CHO cells.

Eukaryotic cell lines

Policy information about cell lines

Cell line source(s)	HL-60 (ATCC CCL-240)

Authentication	HL-60 cells were recently acquired from ATCC. The cells were checked using flow cytometry for surface marker CD11B.
Mycoplasma contamination	HL-60 cell line tested negative for mycoplasma contamination.
Commonly misidentified lines (See ICLAC register)	No commonly misidentified cell lines were used.

Animals and other organisms

Policy information about studies involving animals; ARRIVE guidelines recommended for reporting animal research

Laboratory animals	Species: mus musculus<br>Strain: C57/BL6<br>Age: 6-10 weeks old<br>Sex: Male and female<br>All mice were group-housed on a 12h light/dark cycle, the environmental conditions were maintained thermostatically between 18C-23C with 40-60% humidity, fed ad libitum and had free access to drinking water.<br><br>Species: danio rerio<br>Strain: AB<br>Age: infected at 2 days post fertilization (dpf), monitored for 4 days after<br>Sex: not determined until 21-23 dpf
Wild animals	The study did not include wild animals.
Field-collected samples	The study did not include field-collected samples.
Ethics oversight	Mouse experiments were approved by the University of Wisconsin-Madison Institutional Animal Care and Use Committee or the Boston Children's Hospital Institutional Animal Care and Use Committee.

Note that full information on the approval of the study protocol must also be provided in the manuscript.

Human research participants

Policy information about studies involving human research participants

Population characteristics	Blood donors were healthy men and women between the ages of 20-40 years old.
Recruitment	Participants were recruited via email resulting in no explicit bias.
Ethics oversight	University of Wisconsin Institutional Review Board

Note that full information on the approval of the study protocol must also be provided in the manuscript.

Flow Cytometry

Plots

Confirm that:

- [x] The axis labels state the marker and fluorochrome used (e.g. CD4-FITC).
- [x] The axis scales are clearly visible. Include numbers along axes only for bottom left plot of group (a 'group' is an analysis of identical markers).
- [x] All plots are contour plots with outliers or pseudocolor plots.
- [x] A numerical value for number of cells or percentage (with statistics) is provided.

Methodology

Sample preparation	Cells were incubated with surface marker stains and Ghost Dye Violet 450 (Tondo Biosciences) for cell viablity measurements for 30 minutes. Then the cells were washed with PBS and fixed with 0.4% paraformaldehyde.
Instrument	MACs Quant 10 Analyzer (Miltenyi Biotec)
Software	FlowJo10.4 (Tree Star)
Cell population abundance	All live cells (>90%, >100,000 cells in each sample) were analyzed.

Gating strategy

Cells with very low FSC and SSC were gated out. Dead cells (Ghost Dye Violet 450 positive) were then gated out. The median CD11B+ CD15+ (human peripheral blood neutrophils) or CD11B+ (HL-60 cells) signal of the remaining population was determined. Single color controls of CD11B and CD15 were used for compensation

☒ Tick this box to confirm that a figure exemplifying the gating strategy is provided in the Supplementary Information.
