# Supplementary material for: Switching to the cyclic pentose phosphate pathway powers the oxidative burst in activated neutrophils
Source: Nat Metab. 2022 Mar 28;4(3):389–403. doi: 10.1038/s42255-022-00550-8 (PMC8964420; doi:10.1038/s42255-022-00550-8)

Full Western Blot image of Extended Data Figure 5b

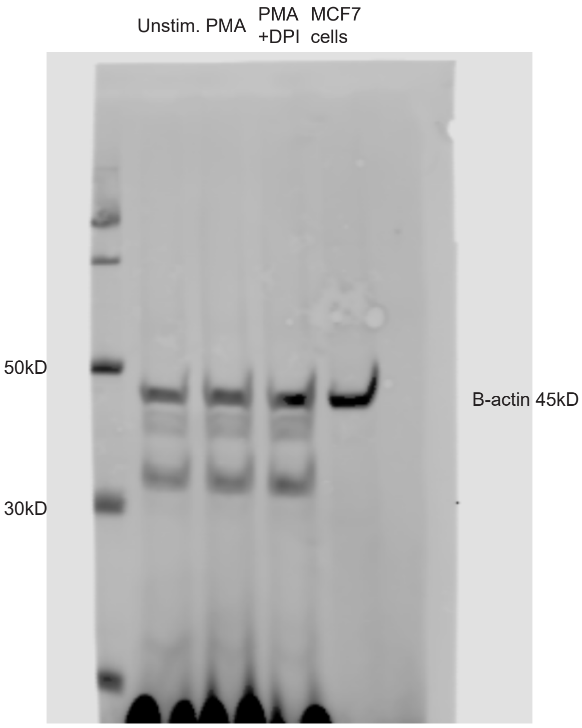

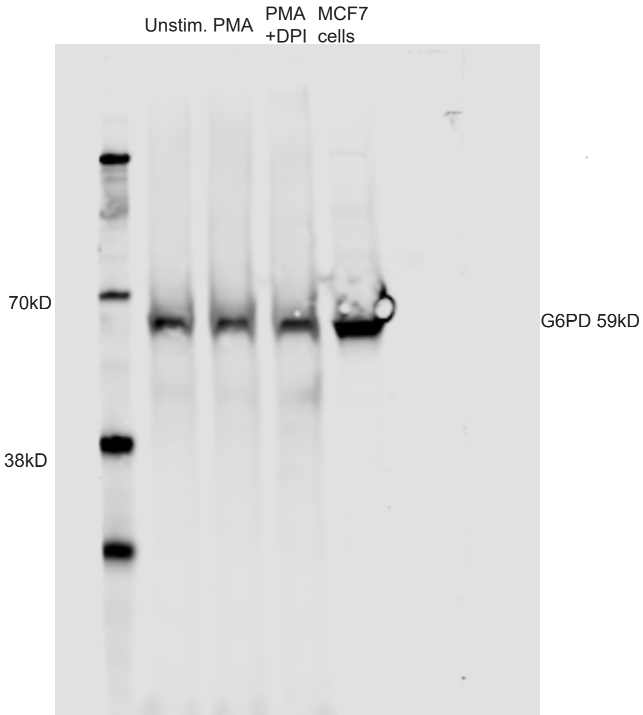

Full Western Blot image of Extended Data Figure 5g

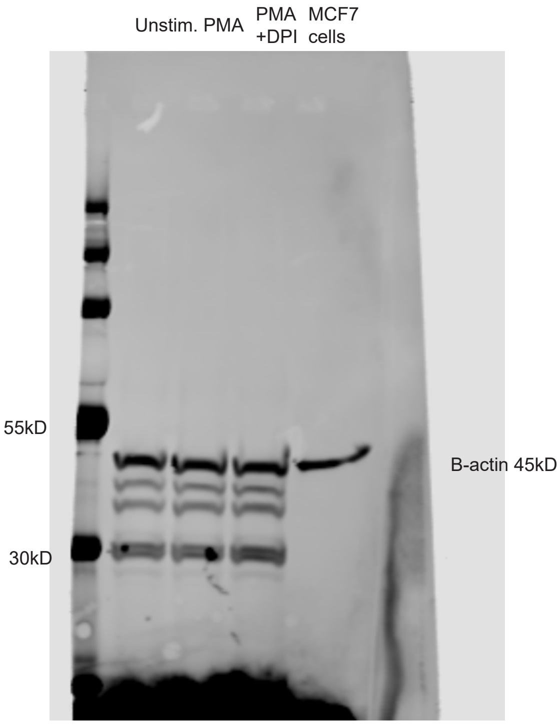

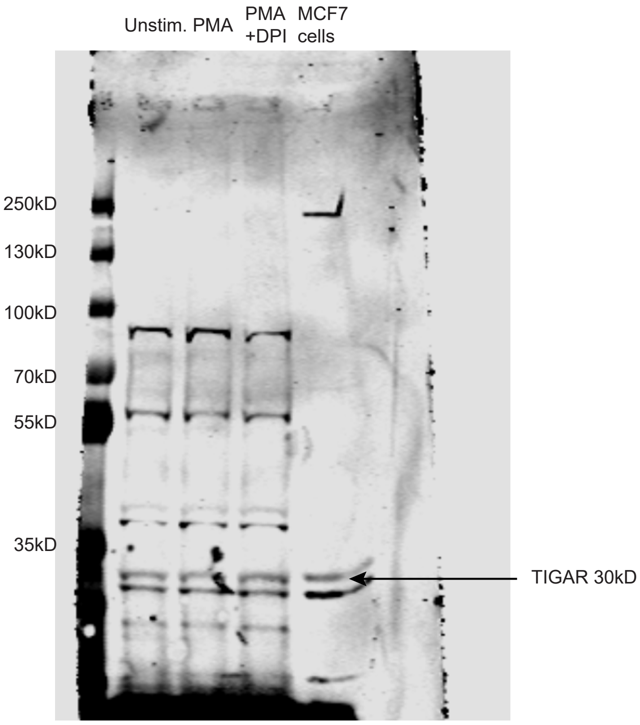

Full Western Blot image of G6PD (replicate experiment)

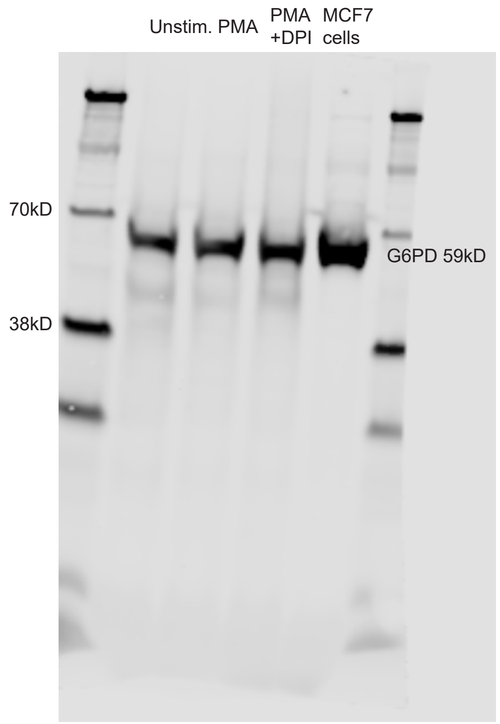

Full Western Blot image of TIGAR (replicate experiment)

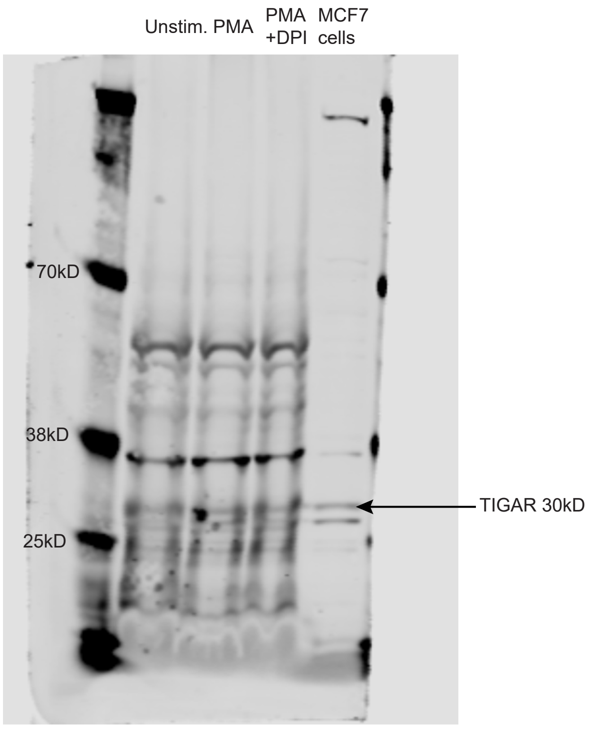

Supplement: Source Data Extended Data Fig. 5 — Unprocessed immunoblots. [file 42255_2022_550_MOESM16_ESM.pdf]
